# Supplementary material for: Computational Metabolomics Tools Reveal Subarmigerides, Unprecedented Linear Peptides from the Marine Sponge Holobiont Callyspongia subarmigera
Source: Mar Drugs. 2022 Oct 27;20(11):673. doi: 10.3390/md20110673 (PMC9696656; doi:10.3390/md20110673)

marine drugs

Article

Computational Metabolomics Tools Reveal Subarmigerides, Unprecedented Linear Peptides from the Marine Sponge Holobiont *Callyspongia subarmigera*

Andrea Castaldi,[1a] Roberta Teta,[2a] Germana Esposito,[2] Mehdi A. Beniddir,[3*] Nicole J. de Voogd,[4,5] Sébastien Duperron,[1] Valeria Costantino,[2*] and Marie-Lise Bourguet-Kondracki[1*]

1 *Molécules de Communication et Adaptation des Microorganismes, UMR 7245 CNRS, Muséum National d'Histoire Naturelle, 57 rue Cuvier (CP54), 75005 Paris, France.*
2 *The Blue Chemistry Lab Group, Dipartimento di Farmacia, Università degli Studi di Napoli Federico II, via D. Montesano 49, 80131 Napoli, Italy*
3 *Équipe "Chimie des substances naturelles" BioCIS, CNRS, Université Paris-Saclay, 17 avenue des Sciences, 91400 Orsay, France.*
4 *Naturalis Biodiversity Center, PO Box 9517, 2300 RA Leiden, The Netherlands*
5 *Institute of Environmental Sciences, Leiden University, Einsteinweg 2, 2333 CC Leiden, The Netherlands*
[a] *Equally contribution*
* *Author to whom correspondence should be addressed*

Figure S1: A) The molecular networking obtained through the LC-MS/MS analysis of the 33 extracts of Haplosclerida sponge collection. B) Zoom view of the discriminant cluster of the *Callyspongia* genus.

Figure S2: A) The molecular networking obtained through the LC-MS/MS analysis of cyanobacterial strains extracts from Guadeloupe's mangroves. B) Zoom view of the peptide.

Figure S3: ^{1}H-NMR spectrum of subarmigeride A (**1**) (600 MHz, DMSO-*d6*).

Figure S4: DEPTQ-NMR spectrum of subarmigeride A (**1**) (150 MHz, DMSO-*d6*).

Figure S5: COSY-NMR spectrum of subarmigeride A (**1**) (600 MHz, DMSO-*d6*).

Figure S6: TOCSY-NMR spectrum of subarmigeride A (**1**) (600 MHz, DMSO-*d6*).

Figure S7: HSQC-NMR spectrum of subarmigeride A (**1**) (600 MHz, DMSO-*d6*).

Figure S8: HMBC-NMR spectrum of subarmigeride A (**1**) (600 MHz, DMSO-*d6*).

Figure S9: NOESY-NMR spectrum of subarmigeride A (**1**) (600 MHz, DMSO-*d6*).

Figure S10: Fragmentations pattern and positive ion mode high-resolution ESI MS/MS spectrum for subarmigeride A (**1**) (*m/z* 857.4914 $[M + H]^+$).

Figure S11: Marfey's analysis in positive ion mode high-resolution ESI mass spectrum of subarmigeride A (**1**).

Figure S12: Fragmentations pattern and positive ion mode high-resolution ESI MS/MS spectrum for subarmigeride B (**2**) (*m/z* 823.5078 $[M + H]^+$).

Figure S13: Fragmentations pattern and positive ion mode high-resolution ESI MS/MS spectrum for subarmigeride C (**3**) (*m/z* 839.5024 $[M + H]^+$).

Figure S14: Fragmentations pattern and positive ion mode high-resolution ESI MS/MS spectrum for subarmigeride D (**4**) (*m/z* 829.4962 $[M + H]^+$).

Figure S15: Fragmentations pattern and positive ion mode high-resolution ESI MS/MS spectrum for subarmigeride E (**5**) (*m/z* 767.4807 $[M + H]^+$).

Figure S16: Fragmentations pattern and positive ion mode high-resolution ESI MS/MS spectrum for subarmigeride F (**6**) (*m*/*z* 768.4650 $[M + H]^+$).
Figure S17: Fragmentations pattern and positive ion mode high-resolution ESI MS/MS spectrum for subarmigeride G (**7**) (*m*/*z* 938.5698 $[M + H]^+$).
Figure S18: Fragmentations pattern and positive ion mode high-resolution ESI MS/MS spectrum for subarmigeride H (**8**) (*m*/*z* 795.4763 $[M + H]^+$).
Figure S19: Comparison of MS/MS spectra of the feature *m*/*z* 857.4920 at 6.036 min in the cyanobacterial strain PMC 1052.18 (*Spirulina* sp.) from a mangrove in Guadeloupe. (A) and the feature *m*/*z* 857.4909 at 5.934 min in the marine sponge *C. subarmigera* (B). Comparison of extracted ion chromatograms for *m*/*z* 857.4912 (tolerance 10 ppm) from the crude extracts of the cyanobacterial strain PMC 1052.18 (C) and the marine sponge *C. subarmigera* (D).
Figure S20: Helically coiled morphology of *Spirulina* sp. PMC 1052.18.
Figure S21: Molecular networks obtained using the Feature-Based Molecular Network workflow on GNPS (https://gnps.ucsd.edu/ProteoSAFe/status.jsp?task=8a40068370b44e21855c1e14647ff23a).
Table S1: Product ion spectra data for subarmigeride B (**2**) (*m*/*z* 823.5078 $[M + H]^+$).
Table S2: Product ion spectra data for subarmigeride C (**3**) (*m*/*z* 839.5024 $[M + H]^+$).
Table S3: Product ion spectra data for subarmigeride D (**4**) (*m*/*z* 829.4962 $[M + H]^+$).
Table S4: Product ion spectra data for subarmigeride E (**5**) (m/z 767.4807 $[M + H]^+$).
Table S5: Product ion spectra data for subarmigeride F (**6**) (m/z 768.4650 $[M + H]^+$).
Table S6: Product ion spectra data for subarmigeride G (**7**) (m/z 938.5698 $[M + H]^+$).
Table S7: Product ion spectra data for subarmigeride H (**8**) (m/z 795.4763 $[M + H]^+$).

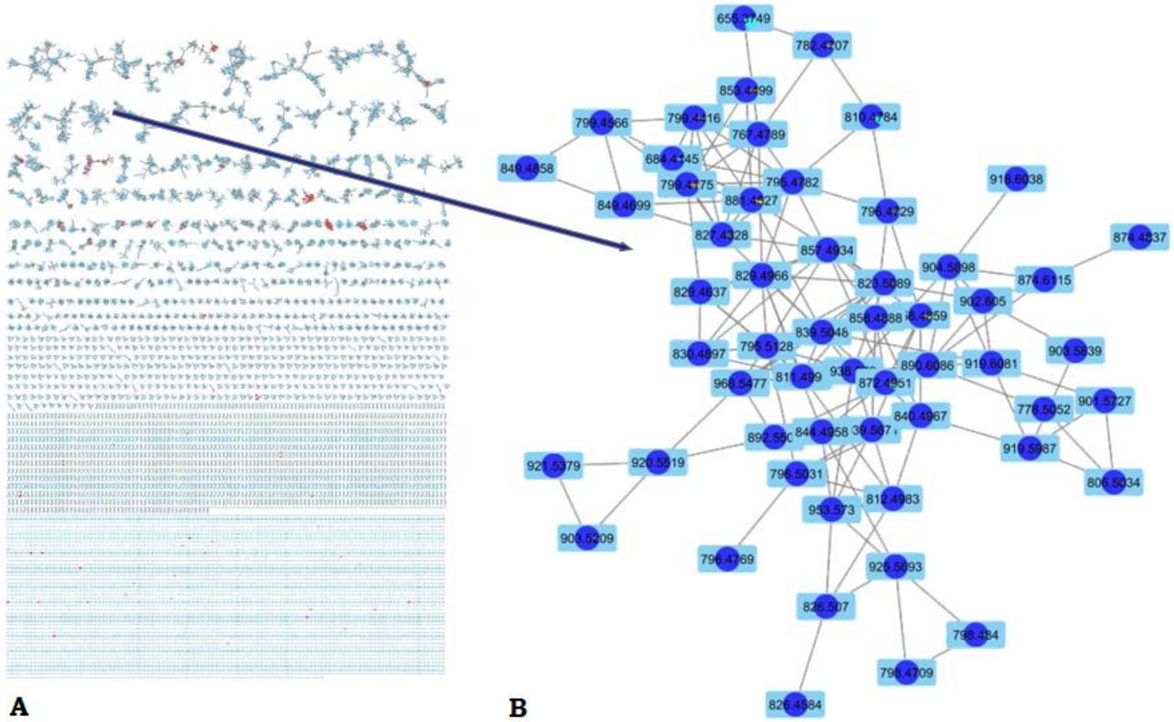

Figure S1: A) The molecular networking obtained through the LC-MS/MS analysis of the 33 extracts of Haplosclerida sponge collection. B) Zoom view of the discriminant cluster of the *Callyspongia* genus.

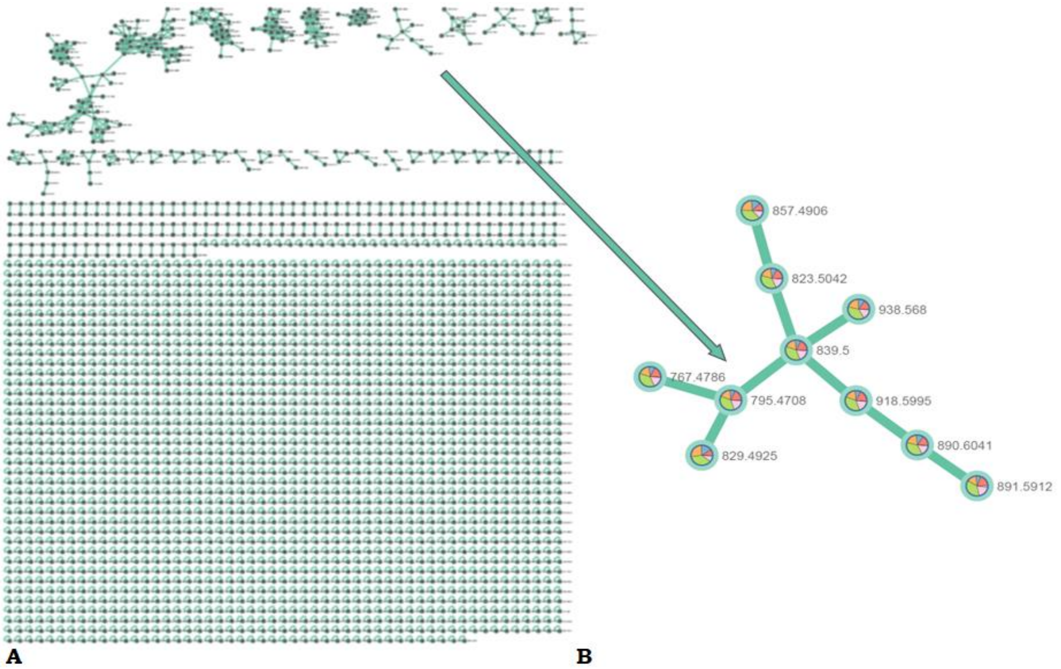

Figure S2: A) The molecular networking obtained through the LC-MS/MS analysis of cyanobacterial strains extracts from Guadeloupe’s mangroves. B) Zoom view of the peptide

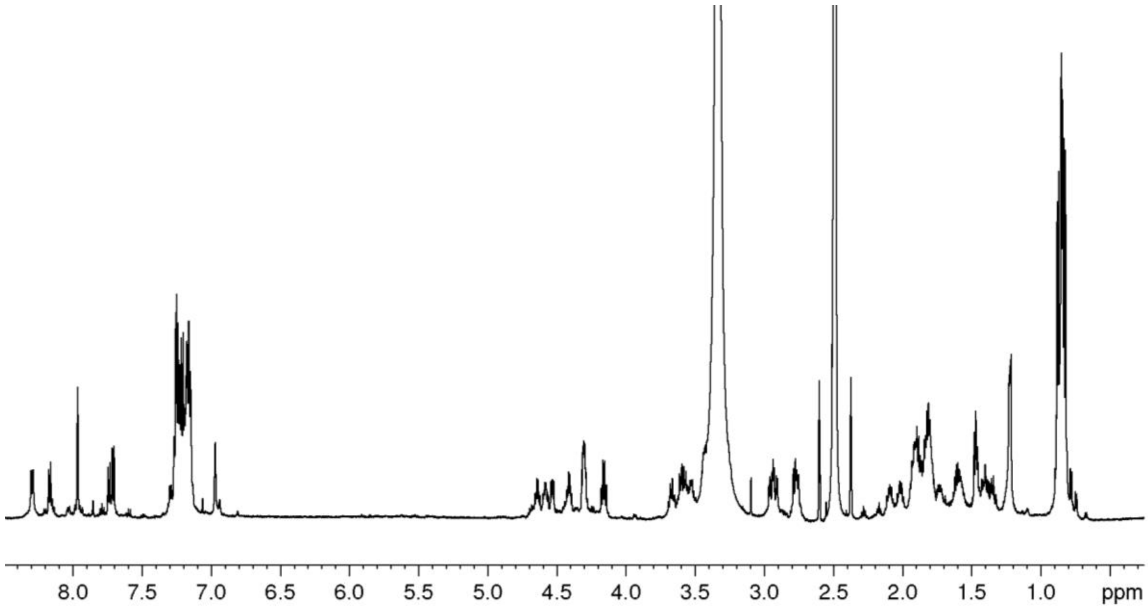

Figure S3: ^{1}H-NMR spectrum of subarmigeride A (**1**) (600 MHz, DMSO-*d6*).

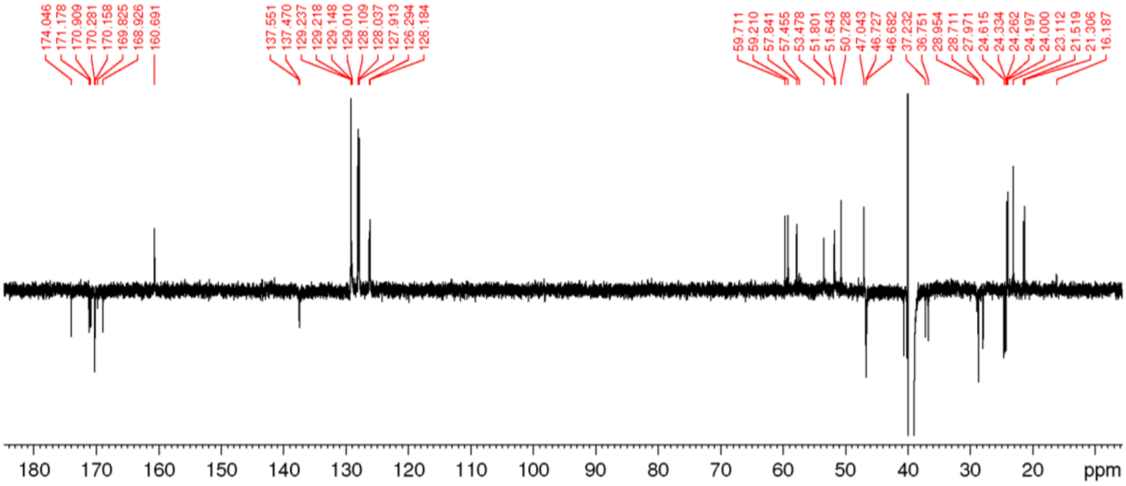

Figure S4: DEPTQ-NMR spectrum of subarmigeride A (**1**) (150 MHz, DMSO-*d6*).

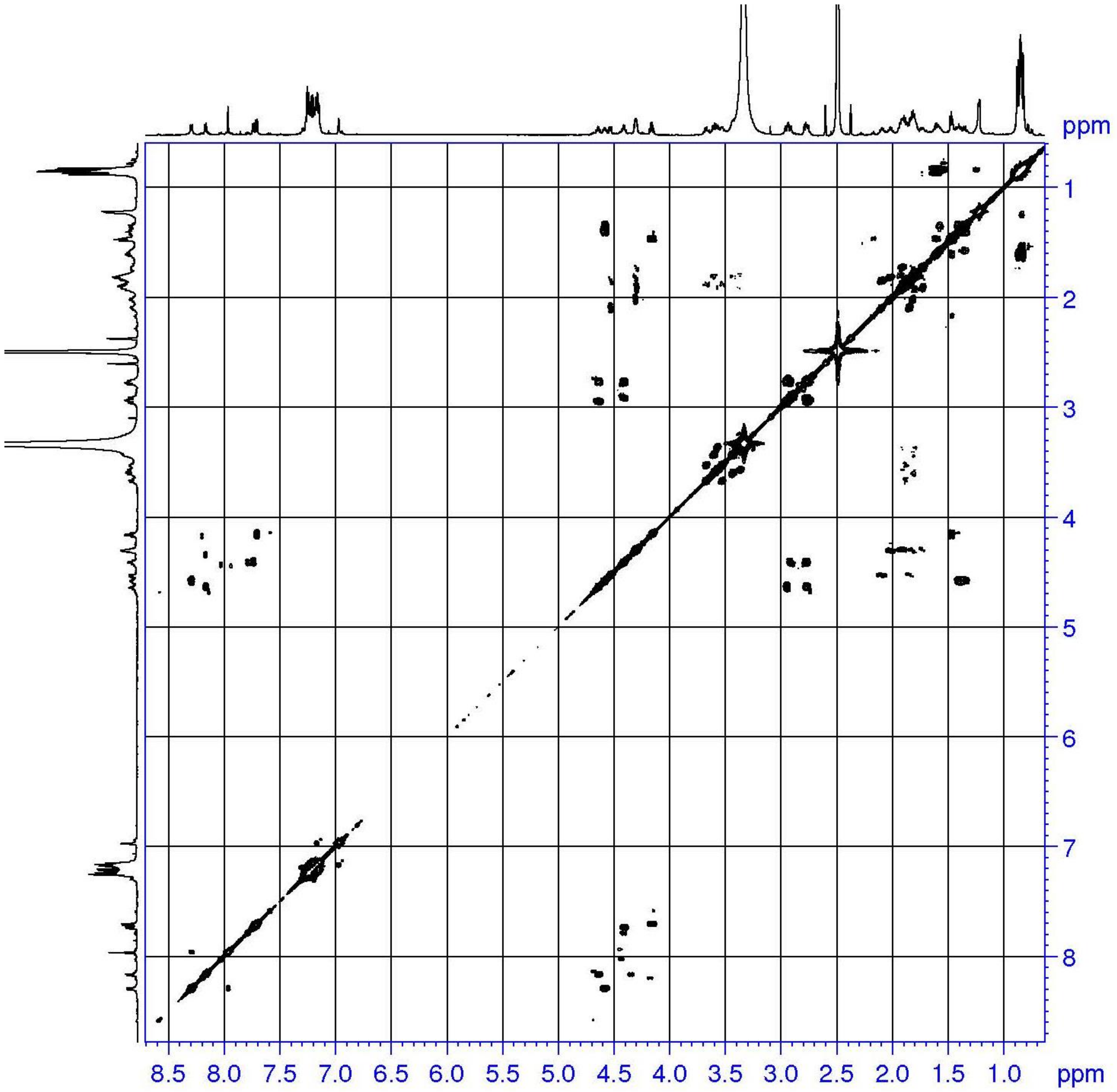

Figure S5: COSY-NMR spectrum of subarmigeride A (**1**) (600 MHz, DMSO-*d6*).

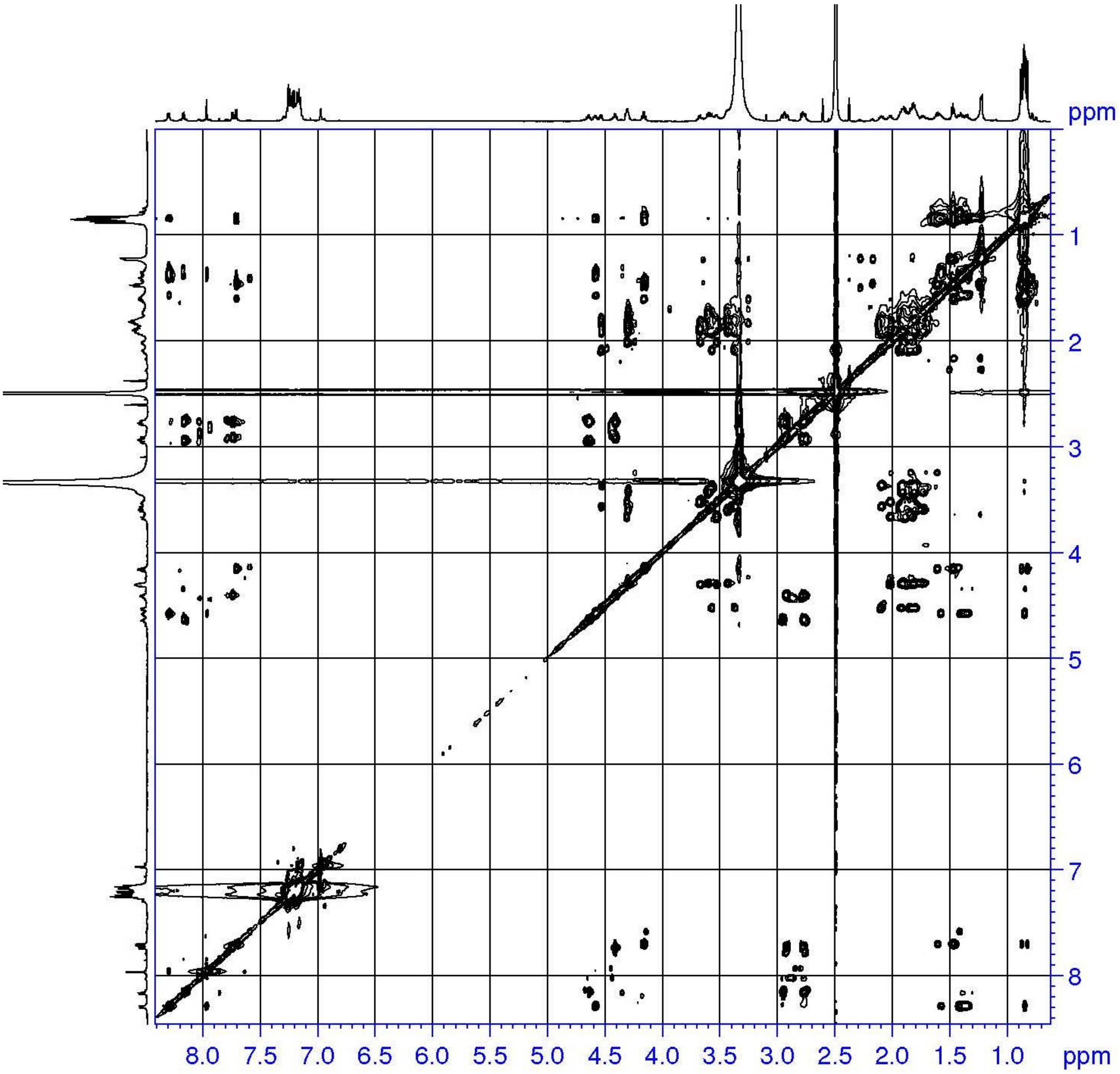

Figure S6: TOCSY-NMR spectrum of subarmigeride A (**1**) (600 MHz, DMSO-*d6*).

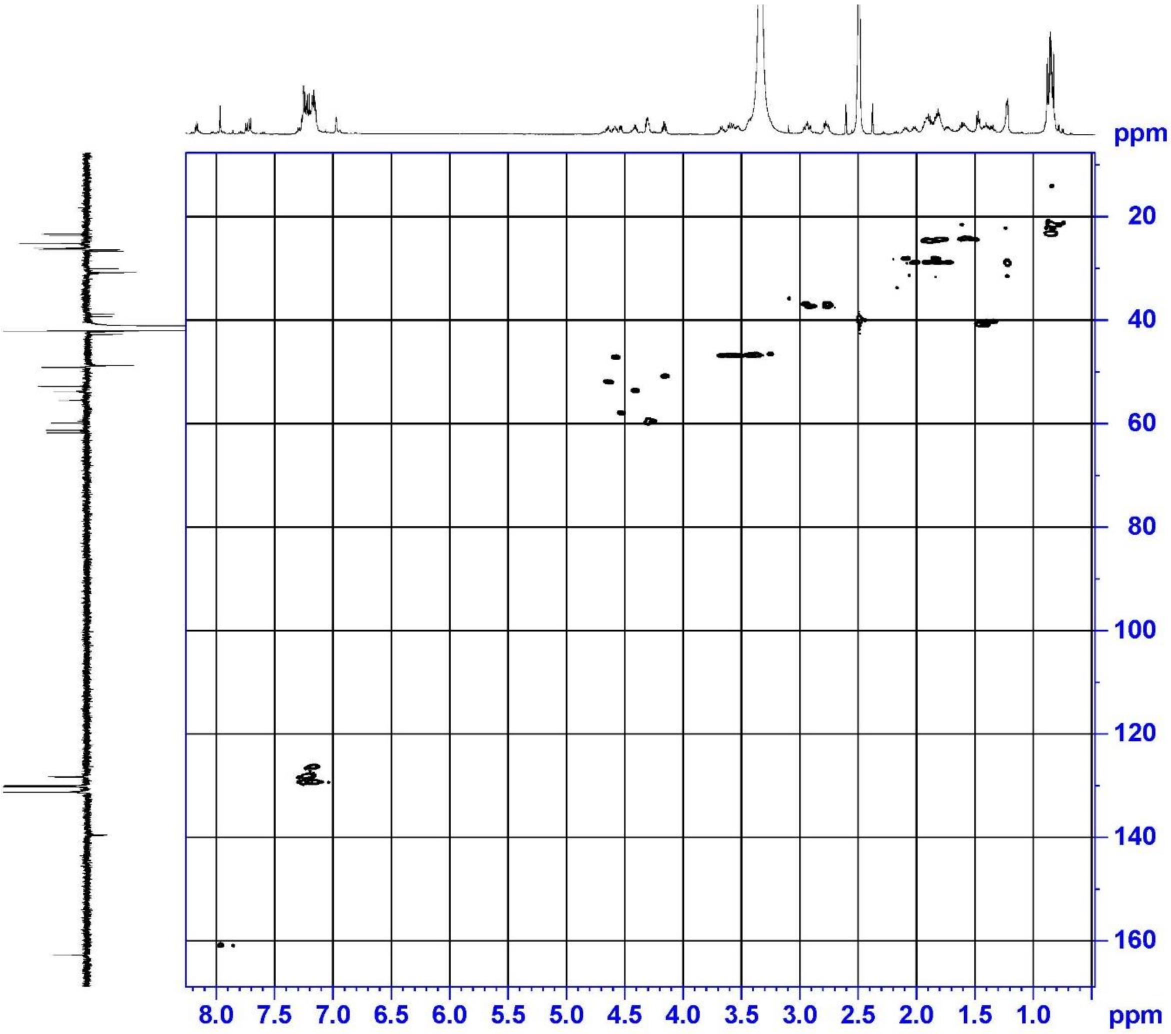

Figure S7: HSQC-NMR spectrum of subarmigeride A (**1**) (600 MHz, DMSO-*d6*).

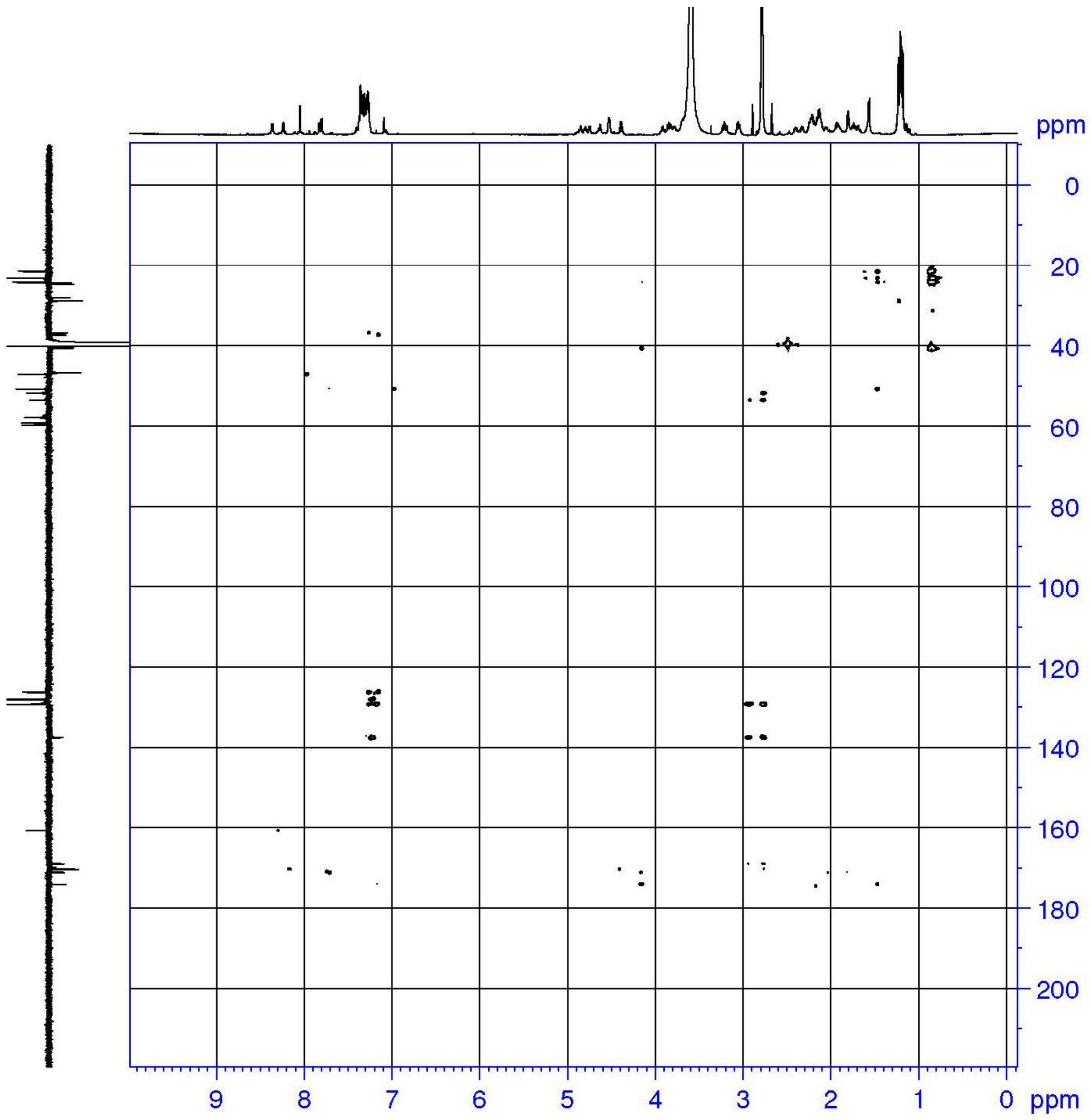

Figure S8: HMBC-NMR spectrum of subarmigeride A (**1**) (600 MHz, DMSO-*d6*).

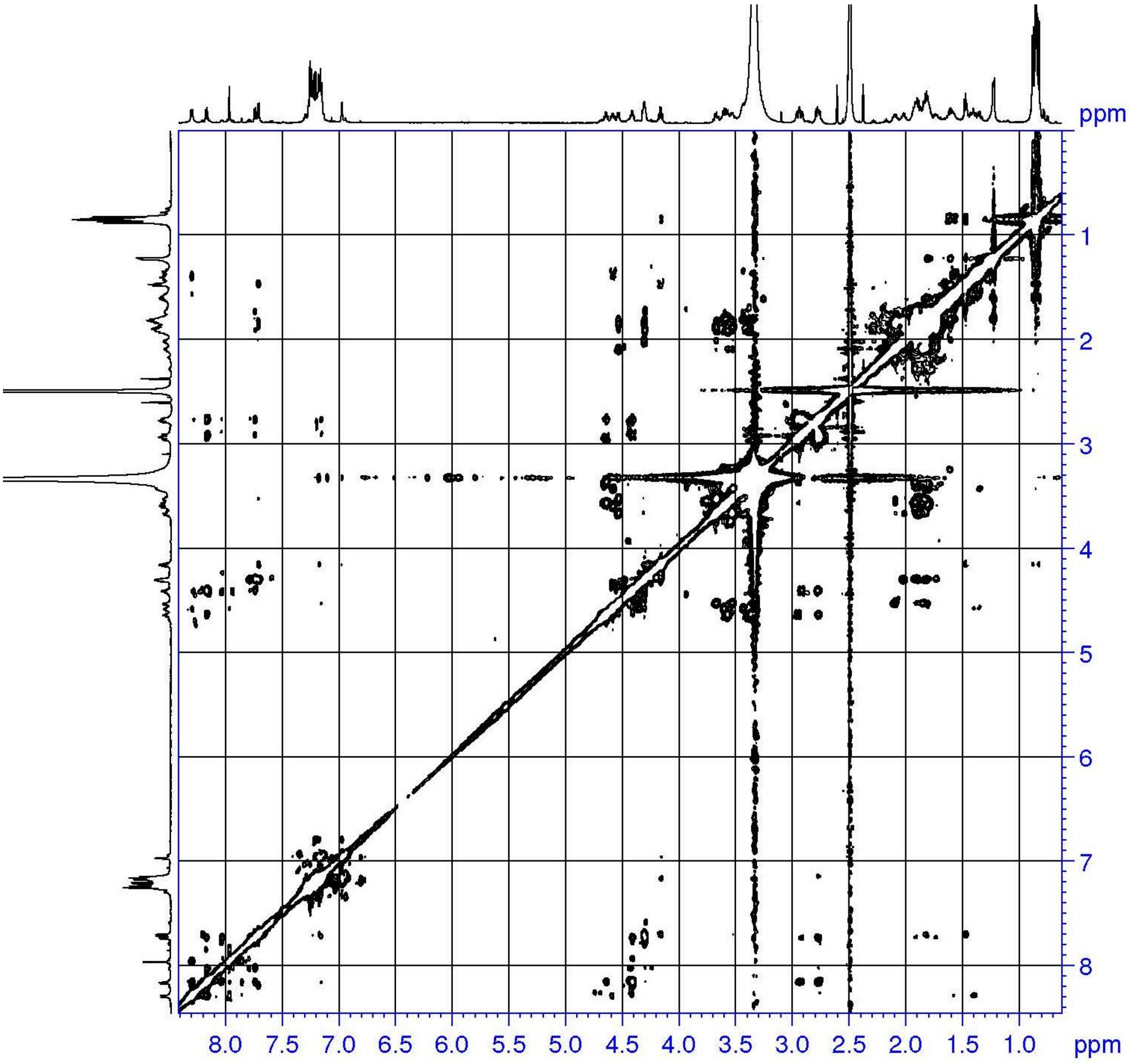

Figure S9: NOESY-NMR spectrum of subarmigeride A (**1**) (600 MHz, DMSO-*d6*).

Figure S10: Fragmentations pattern and positive ion mode high-resolution ESI MS/MS spectrum for subarmigeride A (**1**) (*m/z* 857.4914 [M + H]⁺).

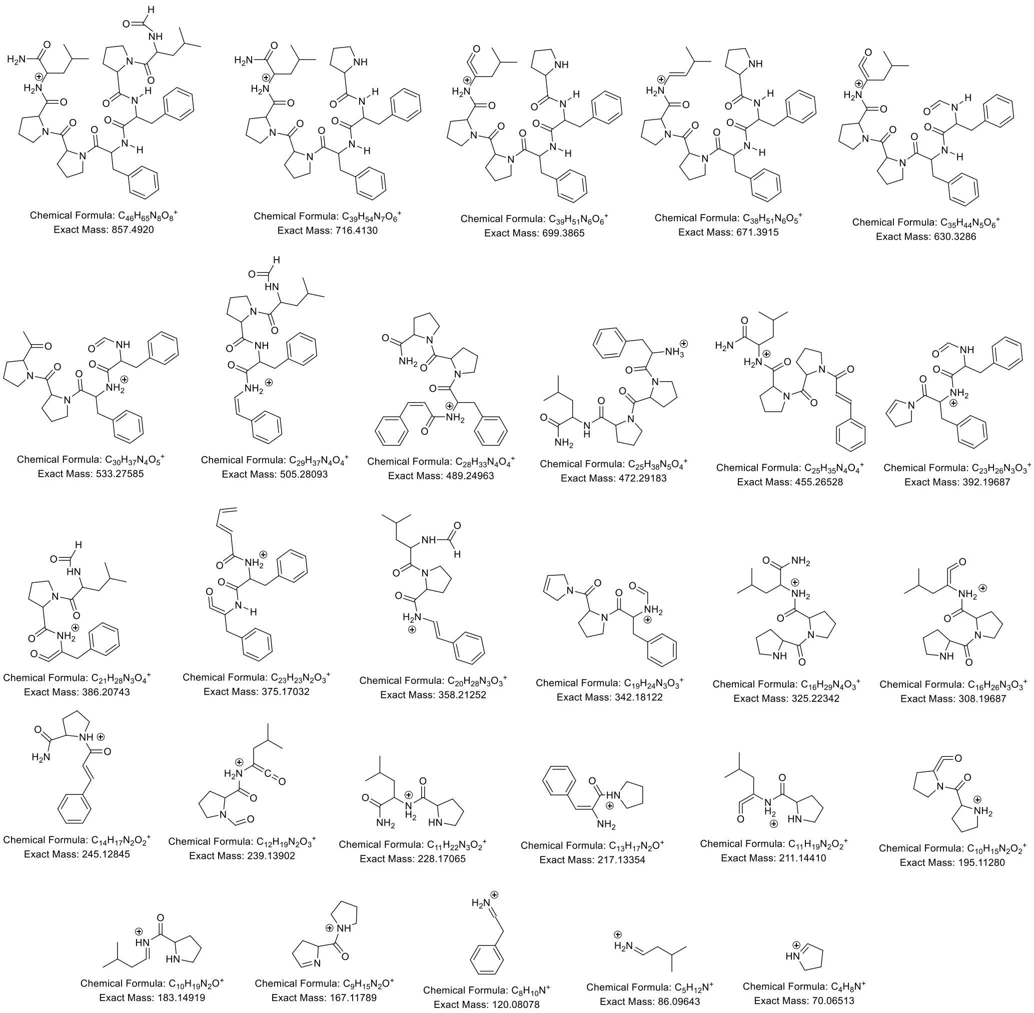

Acquisition Parameter

Source Type	ESI	Ion Polarity	Positive	Set Nebulizer	2.4 Bar
Focus	Not active	Set Capillary	3500 V	Set Dry Heater	200 °C
Scan Begin	50 m/z	Set End Plate Offset	-500 V	Set Dry Gas	8.0 l/min
Scan End	1300 m/z	Set Charging Voltage	2000 V	Set Divert Valve	Waste
		Set Corona	0 nA	Set APCI Heater	0 °C

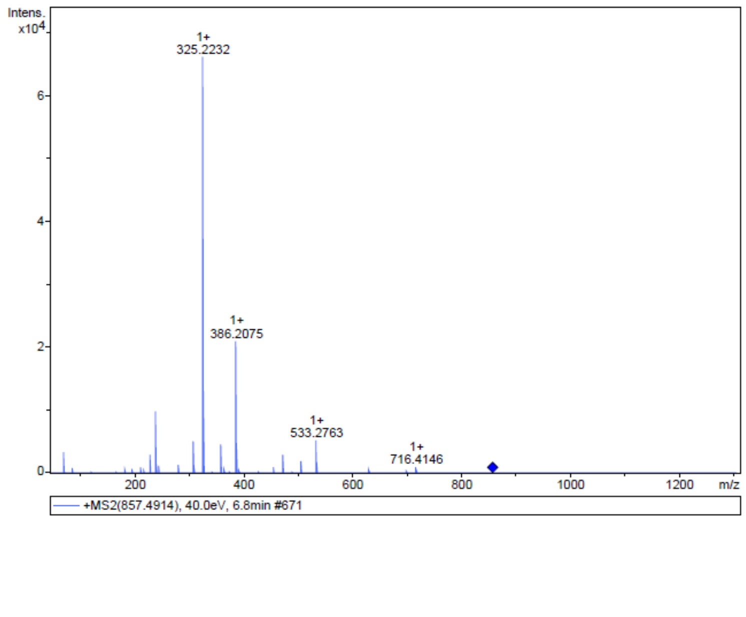

Figure S11: Marfey's analysis in positive ion mode high-resolution ESI mass spectrum of subarmigeride A (**1**).

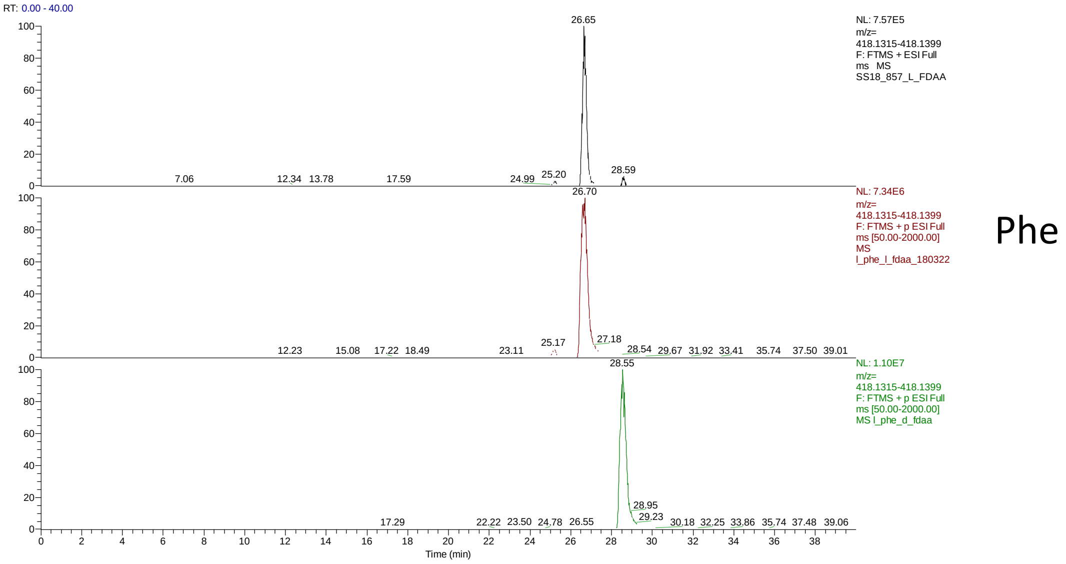

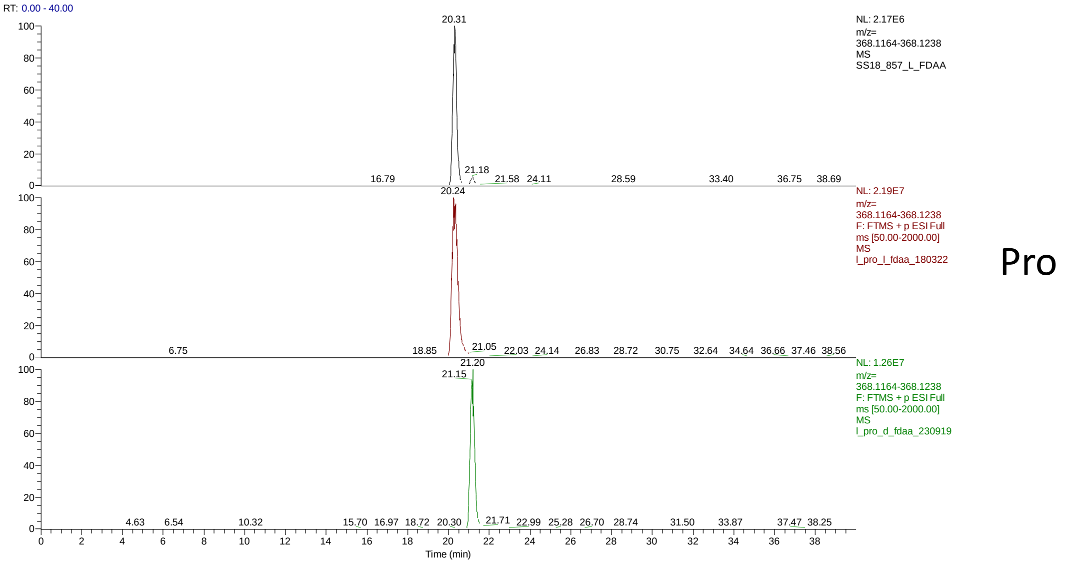

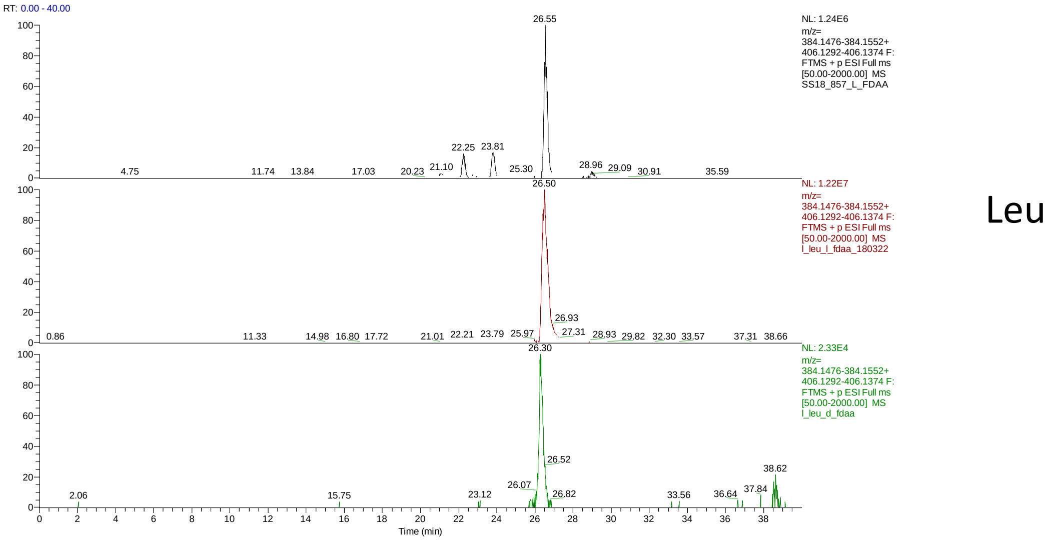

Table S1: Product ion spectra data for subarmigeride B (**2**) (m/z 823.5078 [M + H]$^+$)

Product ion Assignment	(*m/z*)	Error, pm	Molecular Formula
CHO-Ile/Leu- Ile/Leu-Pro-Phe-Pro-Pro-Ile/Leu-NH_2 + H^+	823.5076	-0.2	$C_{43}H_{67}N_8O_8$
CHO-Ile/Leu- Ile/Leu-Pro-Phe-Pro-Pro + H^+	665.4012	1.4	$C_{36}H_{53}N_6O_6$
CHO-Ile/Leu- Ile/Leu-Pro-Phe-Pro + H^+	596.3429	2.2	$C_{32}H_{46}N_5O_6$
CHO-Ile/Leu-Ile/Leu-Pro-Phe + H^+	499.2911	0.9	$C_{27}H_{39}N_4O_5$
Phe-Pro-Pro-Ile/Leu + H^+	472.2921	-0.6	$C_{25}H_{38}N_5O_4$
Phe-Pro-Pro-Ile/Leu + H^+	427.2690	3.2	$C_{24}H_{35}N_4O_3$
CHO-Ile/Leu-Ile/Leu-Pro + H^+	352.2226	1.3	$C_{18}H_{30}N_3O_4$
Ile/Leu-Pro-Phe + H^+	330.2166	3	$C_{19}H_{28}N_3O_2$
Pro-Pro-Ile/Leu-NH_2 + H^+	325.2231	1.1	$C_{16}H_{29}N_4O_3$
Pro-Pro-Ile/Leu + H^+	280.2022	-1	$C_{15}H_{26}N_3O_2$
Phe-Pro-NH_2 + H^+	245.1276	3.6	$C_{14}H_{17}N_2O_2$
Pro-Ile/Leu + H^+	239.1387	1.2	$C_{12}H_{19}N_2O_3$
Pro-Ile/Leu-NH_2 + H^+	228.1703	1.4	$C_{11}H_{22}N_3O_2$
Phe-Pro + H^+	217.1328	3.2	$C_{13}H_{17}N_2O$
Pro-Ile/Leu + H^+	211.1442	-0.7	$C_{11}H_{19}N_2O_2$
Pro-Pro + H^+	195.1129	-0.6	$C_{10}H_{15}N_2O_2$
Pro-Ile/Leu + H^+	183.1488	2	$C_{10}H_{19}N_2O$
Pro-Pro + H^+	167.1179	0	$C_9H_{15}N_2O$
Ile/Leu immonium fragment + H^+	86.0961	3.8	$C_5H_{12}N$
Pro immonium fragment + H^+	70.0647	6.5	C_4H_8N

Figure S12: Fragmentations pattern and positive ion mode high-resolution ESI MS/MS spectrum for subarmigeride B (**2**) (*m/z* 823.5078 [M + H]⁺)

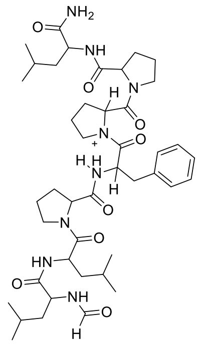

Chemical Formula: $C_{43}H_{67}N_8O_8^+$
Exact Mass: 823.5076

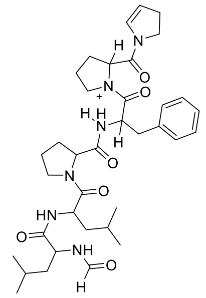

Chemical Formula: $C_{36}H_{53}N_6O_6^+$
Exact Mass: 665.4021

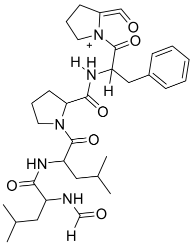

Chemical Formula: $C_{32}H_{46}N_5O_6^+$
Exact Mass: 596.3443

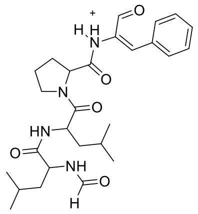

Chemical Formula: $C_{27}H_{39}N_4O_5^+$
Exact Mass: 499.2915

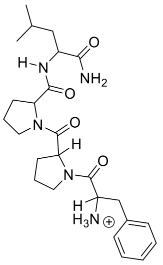

Chemical Formula: $C_{25}H_{38}N_5O_4^+$
Exact Mass: 472.2918

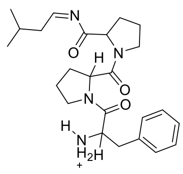

Chemical Formula: $C_{24}H_{35}N_4O_3^+$
Exact Mass: 427.2704

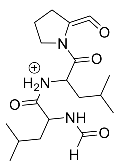

Chemical Formula: $C_{18}H_{30}N_3O_4^+$
Exact Mass: 352.2231

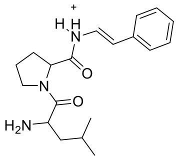

Chemical Formula: $C_{19}H_{28}N_3O_2^+$
Exact Mass: 330.2176

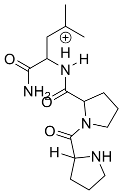

Chemical Formula: $C_{16}H_{29}N_4O_3^+$
Exact Mass: 325.2234

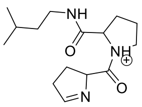

Chemical Formula: $C_{15}H_{26}N_3O_2^+$
Exact Mass: 280.2020

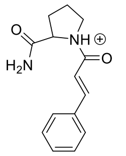

Chemical Formula: $C_{14}H_{17}N_2O_2^+$
Exact Mass: 245.1285

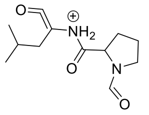

Chemical Formula: $C_{12}H_{19}N_2O_3^+$
Exact Mass: 239.1390

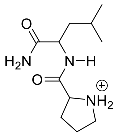

Chemical Formula: $C_{11}H_{22}N_3O_2^+$
Exact Mass: 228.1707

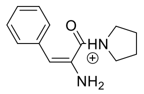

Chemical Formula: $C_{13}H_{17}N_2O^+$
Exact Mass: 217.1335

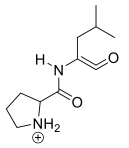

Chemical Formula: $C_{11}H_{19}N_2O_2^+$
Exact Mass: 211.1441

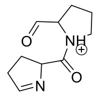

Chemical Formula: $C_{10}H_{15}N_2O_2^+$
Exact Mass: 195.1128

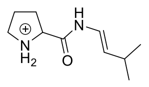

Chemical Formula: $C_{10}H_{19}N_2O^+$
Exact Mass: 183.1492

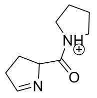

Chemical Formula: $C_9H_{15}N_2O^+$
Exact Mass: 167.1179

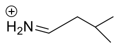

Chemical Formula: $C_5H_{12}N^+$
Exact Mass: 86.0964

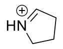

Chemical Formula: $C_4H_8N^+$
Exact Mass: 70.0651

Acquisition Parameter

Source Type	ESI	Ion Polarity	Positive	Set Nebulizer	2.4 Bar
Focus	Not active	Set Capillary	3500 V	Set Dry Heater	200 °C
Scan Begin	50 m/z	Set End Plate Offset	-500 V	Set Dry Gas	8.0 l/min
Scan End	1300 m/z	Set Charging Voltage	2000 V	Set Divert Valve	Waste
		Set Corona	0 nA	Set APCI Heater	0 °C

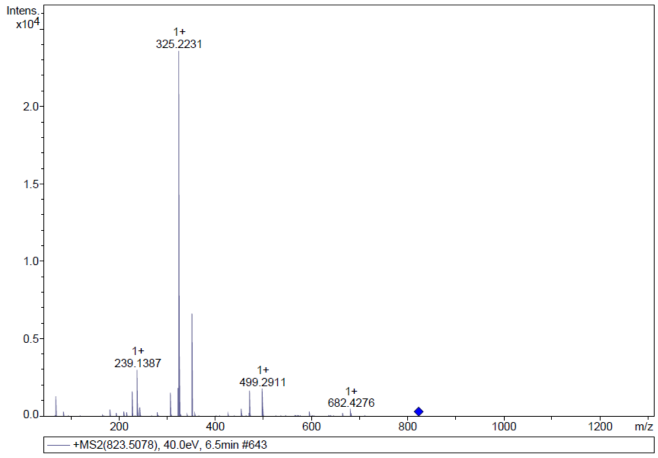

Table S2: Product ion spectra data for subarmigeride C (**3**) (*m*/*z* 839.5024 [M + H]+)

Product ion Assignment	(*m*/*z*)	Error, pm	Molecular Formula
HOOC-Ile/Leu-Pro-Ile/Leu-Phe-Pro-Pro-Ile/Leu-NH_2 + H^+	839.5024	0.2	$C_{43}H_{67}N_8O_9$
HOOC-Ile/Leu-Pro-Ile/Leu-Phe-Pro-Pro + H^+	681.3973	-0.3	$C_{36}H_{53}N_6O_7$
HOOC-Ile/Leu-Pro-Ile/Leu-Phe-Pro + H^+	612.3406	-2.4	$C_{32}H_{46}N_5O_7$
HOOC-Ile/Leu-Pro-Ile/Leu-Phe + H^+	515.2860	0.8	$C_{27}H_{39}N_4O_6$
HOOC-Ile/Leu-Pro-Ile/Leu-Phe + H^+	487.2913	0.3	$C_{26}H_{39}N_4O_5$
Phe-Pro-Pro-Ile/Leu-NH_2 + H^+	472.2920	-0.4	$C_{25}H_{38}N_5O_4$
Phe-Pro-Pro-Ile/Leu-NH_2 + H^+	455.2655	-0.5	$C_{25}H_{35}N_4O_4$
Phe-Pro-Pro-Ile/Leu + H^+	427.2698	1.2	$C_{24}H_{35}N_4O_3$
HOOC-Ile/Leu-Pro-Ile/Leu + H^+	368.2178	0.6	$C_{18}H_{30}N_3O_5$
HOOC-Ile/Leu-Pro-Ile/Leu + H^+	340.2228	1	$C_{17}H_{30}N_3O_4$
Pro-Pro-Ile/Leu-NH_2 + H^+	325.2231	0.8	$C_{16}H_{29}N_4O_3$
Pro-Pro-Ile/Leu + H^+	308.1964	1.4	$C_{16}H_{26}N_3O_3$
Pro-Pro-Ile/Leu + H^+	280.2017	1	$C_{15}H_{26}N_3O_2$
HOOC-Ile/Leu-Pro + H^+	255.1337	0.7	$C_{12}H_{19}N_2O_4$
Phe-Pro + H^+	245.1281	1.6	$C_{14}H_{17}N_2O_2$
Pro-Ile/Leu-NH_2 + H^+	228.1703	1.7	$C_{11}H_{22}N_3O_2$
Phe-Pro + H^+	217.1333	1.2	$C_{13}H_{17}N_2O$
Pro-Ile/Leu + H^+	211.1433	3.6	$C_{11}H_{19}N_2O_2$
Pro-Pro + H^+	195.1126	1	$C_{10}H_{15}N_2O_2$
Pro-Ile/Leu + H^+	183.1489	1.8	$C_{10}H_{19}N_2O$
Pro-Pro + H^+	167.1177	1.1	$C_9H_{15}N_2O$
Ile/Leu immonium fragment + H^+	86.0964	0.9	$C_5H_{12}N$
Pro immonium fragment + H^+	70.0648	4.1	C_4H_8N

Figure S13: Fragmentations pattern and positive ion mode high-resolution ESI MS/MS spectrum for subarmigeride C (**3**) (*m*/*z* 839.5024 [M + H]⁺)

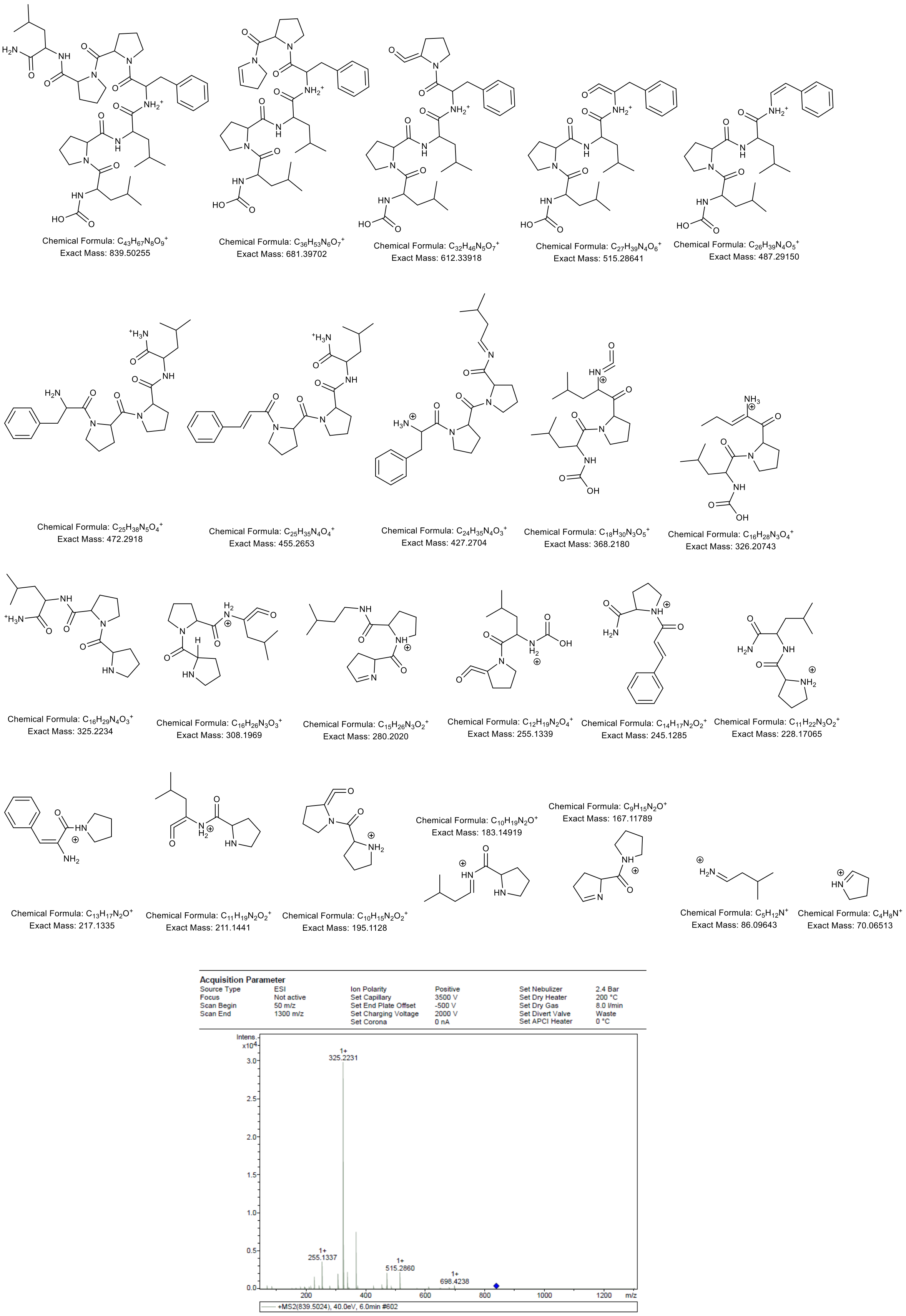

Table S3: Product ion spectra data for subarmigeride D (**4**) (*m/z* 829.4962 [M + H]⁺)

**Product ion Assignment**	**(*m/z*)**	**Error, pm**	**Molecular Formula**
Ile/Leu-Pro-Phe-Phe-Pro-Pro-Ile/Leu-NH_2 + H^+	829.4962	-0.4	$C_{45}H_{65}N_8O_7$
Pro-Phe-Phe-Pro-Pro-Ile/Leu-NH_2 + H^+	716.4143	-1.7	$C_{39}H_{54}N_7O_6$
Pro-Phe-Phe-Pro-Pro-Ile/Leu + H^+	699.3865	-0.1	$C_{39}H_{51}N_6O_6$
Phe-Phe-Pro-Pro-Ile/Leu-NH_2 + H^+	619.3591	1.8	$C_{35}H_{54}N_7O_6$
Phe-Phe-Pro-Pro-Ile/Leu-NH_2 + H^+	602.3342	-0.9	$C_{34}H_{47}N_6O_5$
Pro-Phe-Phe-Pro-Pro + H^+	584.3227	0.7	$C_{34}H_{44}N_5O_5$
Ile/Leu-Pro-Phe-Phe-Pro + H^+	574.3393	-0.9	$C_{34}H_{42}N_5O_4$
Ile/Leu-Pro-Phe-Phe + H^+	505.2812	-0.6	$C_{33}H_{44}N_5O_4$
Phe-Phe-Pro-Pro + H^+	489.2499	-0.6	$C_{29}H_{37}N_4O_4$
Ile/Leu-Pro-Phe-Phe + H^+	477.2859	0.2	$C_{28}H_{33}N_4O_4$
Ile/Leu-Pro-Phe + H^+	472.2920	-0.3	$C_{28}H_{37}N_4O_3$
Phe-Pro-Pro-Ile/Leu + H^+	455.2660	-1.6	$C_{25}H_{38}N_5O_4$
Phe-Phe-Pro + H^+	392.1967	0.5	$C_{25}H_{35}N_4O_4$
Ile/Leu-Pro-Phe + H^+	358.2124	0.3	$C_{23}H_{26}N_3O_3$
Pro-Pro-Ile/Leu-NH_2 + H^+	325.2230	1.2	$C_{20}H_{28}N_3O_3$
Pro-Pro-Ile/Leu + H^+	308.1968	0.4	$C_{16}H_{29}N_4O_3$
Phe-Pro + H^+	245.1281	1.6	$C_{16}H_{26}N_3O_3$
Pro-Ile/Leu-NH_2 + H^+	228.1702	1.9	$C_{14}H_{17}N_2O_2$
Phe-Pro + H^+	217.1327	3.9	$C_{11}H_{22}N_3O_2$
Pro-Ile/Leu + H^+	211.1435	3.1	$C_{13}H_{17}N_2O$
Phe immonium fragment + H^+	120.0809	-0.7	$C_9H_{17}N_2O$
Ile/Leu immonium fragment + H^+	86.0961	3.2	$C_8H_{10}N$
Pro immonium fragment + H^+	70.0649	3.3	$C_5H_{12}N$

Figure S14: Fragmentations pattern and positive ion mode high-resolution ESI MS/MS spectrum for subarmigeride D (**4**) (*m*/*z* 829.4962 [M + H]⁺)

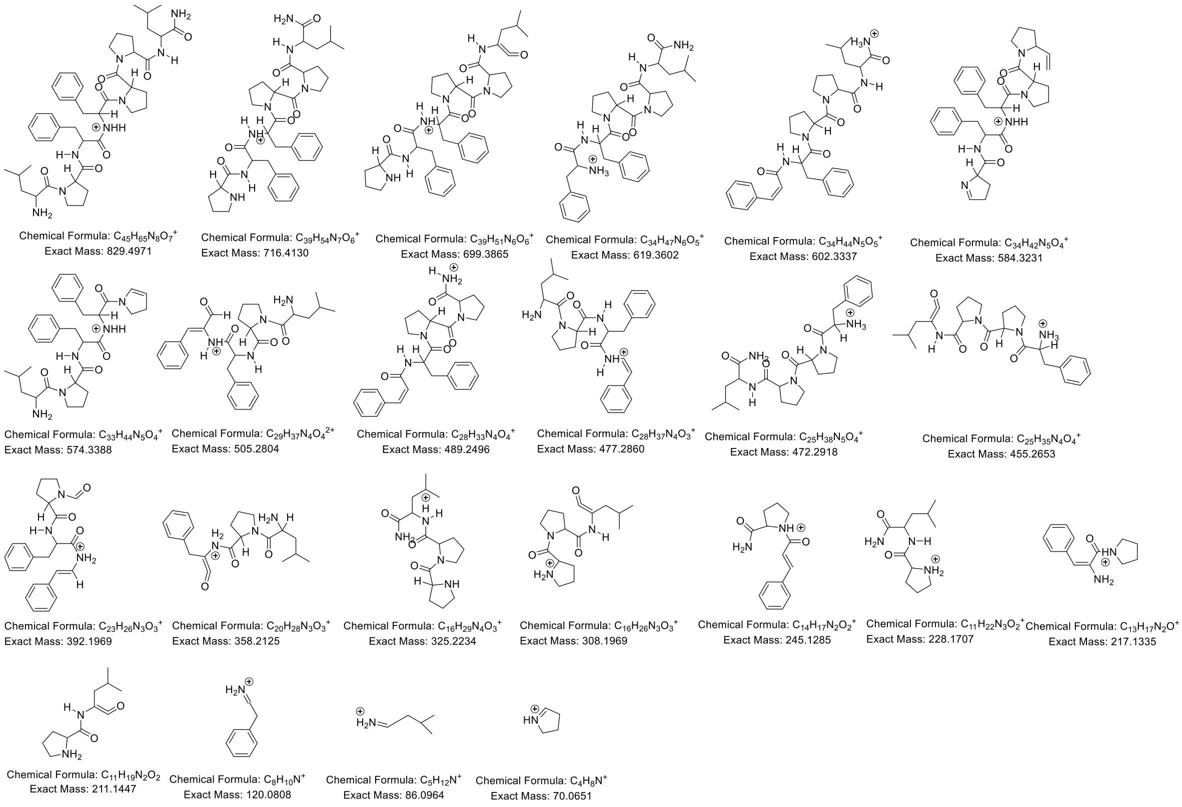

Acquisition Parameter

Source Type	ESI	Ion Polarity	Positive	Set Nebulizer	2.4 Bar
Focus	Not active	Set Capillary	3500 V	Set Dry Heater	200 °C
Scan Begin	50 m/z	Set End Plate Offset	-500 V	Set Dry Gas	8.0 l/min
Scan End	1300 m/z	Set Charging Voltage	2000 V	Set Divert Valve	Waste
		Set Corona	0 nA	Set APCI Heater	0 °C

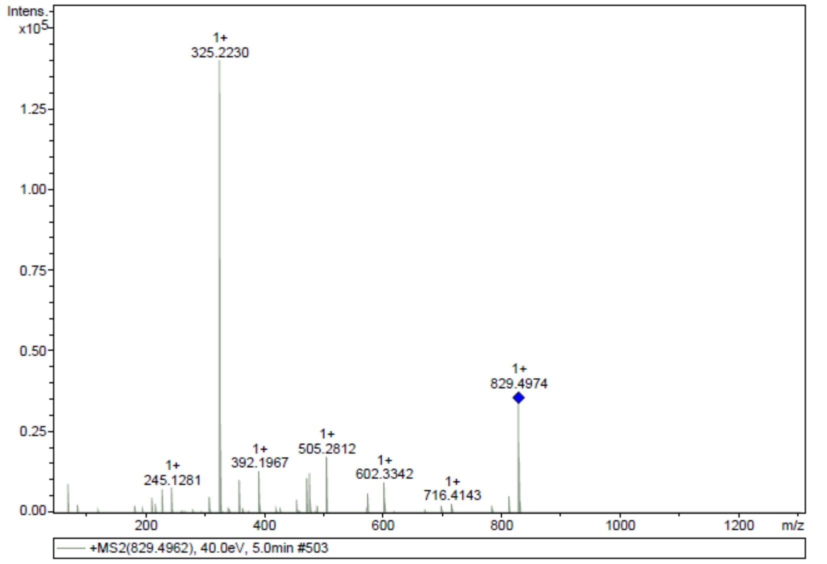

Table S4: Product ion spectra data for subarmigeride E (**5**) (*m/z* 767.4807 [M + H]+)

Product ion Assignment	(*m/z*)	Error, pm	Molecular Formula
Val-Val-Pro-Phe-Pro-Pro-Ile/Leu-NH_2 + H^+	767.4807	0.9	$C_{40}H_{63}N_8O_7$
Val-Val-Pro-Phe-Pro-Pro-Ile/Leu + H^+	750.4557	-1.1	$C_{40}H_{60}N_7O_7$
Val-Val-Pro-Phe-Pro-Pro-Ile/Leu + H^+	722.4602	-0.3	$C_{39}H_{60}N_7O_6$
Val-Pro-Phe-Pro-Pro-Ile/Leu-NH_2 + H^+	668.4136	-0.8	$C_{35}H_{54}N_7O_6$
Val-Pro-Phe-Pro-Pro-Ile/Leu-NH_2 + H^+	651.3864	0.1	$C_{35}H_{51}N_6O_6$
Val-Val-Pro-Phe-Pro-Pro + H^+	637.3710	-0.3	$C_{34}H_{49}N_6O_6$
Val-Pro-Phe-Pro-Pro-Ile/Leu + H^+	623.3914	0.2	$C_{34}H_{51}N_6O_5$
Val-Val-Pro-Phe-Pro-Pro + H^+	609.3763	-0.7	$C_{33}H_{49}N_6O_5$
Pro-Phe-Pro-Pro-Ile/Leu + H^+	554.3330	1.2	$C_{30}H_{44}N_5O_5$
Val-Val-Pro-Phe-Pro + H^+	540.3182	-0.4	$C_{29}H_{42}N_5O_5$
Val-Val-Pro-Phe-Pro + H^+	512.3237	-1.1	$C_{28}H_{42}N_5O_4$
Phe-Pro-Pro-Ile/Leu-NH_2 + H^+	472.2919	-0.2	$C_{25}H_{38}N_5O_4$
Phe-Pro-Pro-Ile/Leu + H^+	455.2652	0.2	$C_{25}H_{35}N_4O_4$
Val-Val-Pro-Phe-Pro + H^+	443.2652	0.2	$C_{24}H_{35}N_4O_4$
Phe-Pro-Pro-Ile/Leu + H^+	427.2699	1.1	$C_{24}H_{35}N_4O_3$
Val-Val-Pro-Phe + H^+	415.2706	-0.5	$C_{23}H_{35}N_4O_3$
Val-Pro-Phe-Pro + H^+	398.2441	-0.8	$C_{23}H_{32}N_3O_3$
Phe-Pro-Pro + H^+	344.1967	0.5	$C_{19}H_{26}N_3O_3$
Phe-Pro-Pro + H^+	342.1814	-0.4	$C_{19}H_{24}N_3O_3$
Pro-Pro-Ile/Leu + H^+	325.2233	0.5	$C_{16}H_{29}N_4O_3$
Phe-Pro-Pro + H^+	316.2022	-0.7	$C_{18}H_{26}N_3O_2$
Pro-Pro-Ile/Leu + H^+	308.1970	-0.4	$C_{16}H_{26}N_3O_3$
Phe-Pro-Pro + H^+	299.1751	1.1	$C_{18}H_{23}N_2O_2$
Val-Val-Pro + H^+	296.1968	0.4	$C_{15}H_{26}N_3O_3$
Val-Val-Pro + H^+	294.1811	0.3	$C_{15}H_{24}N_3O_3$
Pro-Pro-Ile/Leu + H^+	280.2018	0.4	$C_{15}H_{26}N_3O_2$
Pro-Pro-Ile/Leu + H^+	278.1865	-0.8	$C_{15}H_{24}N_3O_2$
Val-Val-Pro + H^+	268.2017	1	$C_{14}H_{26}N_3O_2$
Phe-Pro + H^+	245.1282	0.9	$C_{14}H_{17}N_2O_2$
Pro-Ile/Leu-NH_2 + H^+	228.1702	1.8	$C_{11}H_{22}N_3O_2$
Phe-Pro + H^+	219.1491	0.6	$C_{13}H_{19}N_2O$
Phe-Pro + H^+	217.1332	1.4	$C_{13}H_{17}N_2O$

Val-Val + H^+	214.1550	-0.2	$C_{10}H_{20}N_3O_2$
Pro-Ile/Leu + H^+	211.1437	1.9	$C_{11}H_{19}N_2O_2$
Val-Pro + H^+	197.1279	3	$C_{10}H_{17}N_2O_2$
Pro-Pro + H^+	195.1122	3	$C_{10}H1_5N_2O_2$
Pro-Ile/Leu + H^+	183.1486	3.1	$C_{10}H_{19}N_2O$
Val-Pro + H^+	169.1331	2.6	$C_9H_{17}N_2O$
Pro-Pro + H^+	167.1172	4	$C_9H_{15}N_2O$
Val fragment + H^+	126.0550	-0.1	$C_6H_8NO_2$
Phe immonium fragment + H^+	120.0813	-4.5	$C_8H_{10}N$
Val fragment + H^+	98.0598	2.5	C_5H_8NO
Ile/Leu immonium fragment + H^+	86.0962	2.4	$C_5H_{12}N$
Val immonium fragment + H^+	72.0804	5.6	$C_4H_{10}N$
Pro immonium fragment + H^+	70.0647	6	C_4H_8N

Figure S15: Fragmentations pattern and positive ion mode high-resolution ESI MS/MS spectrum for subarmigeride E (**5**) (*m/z* 767.4807 [M + H]+)

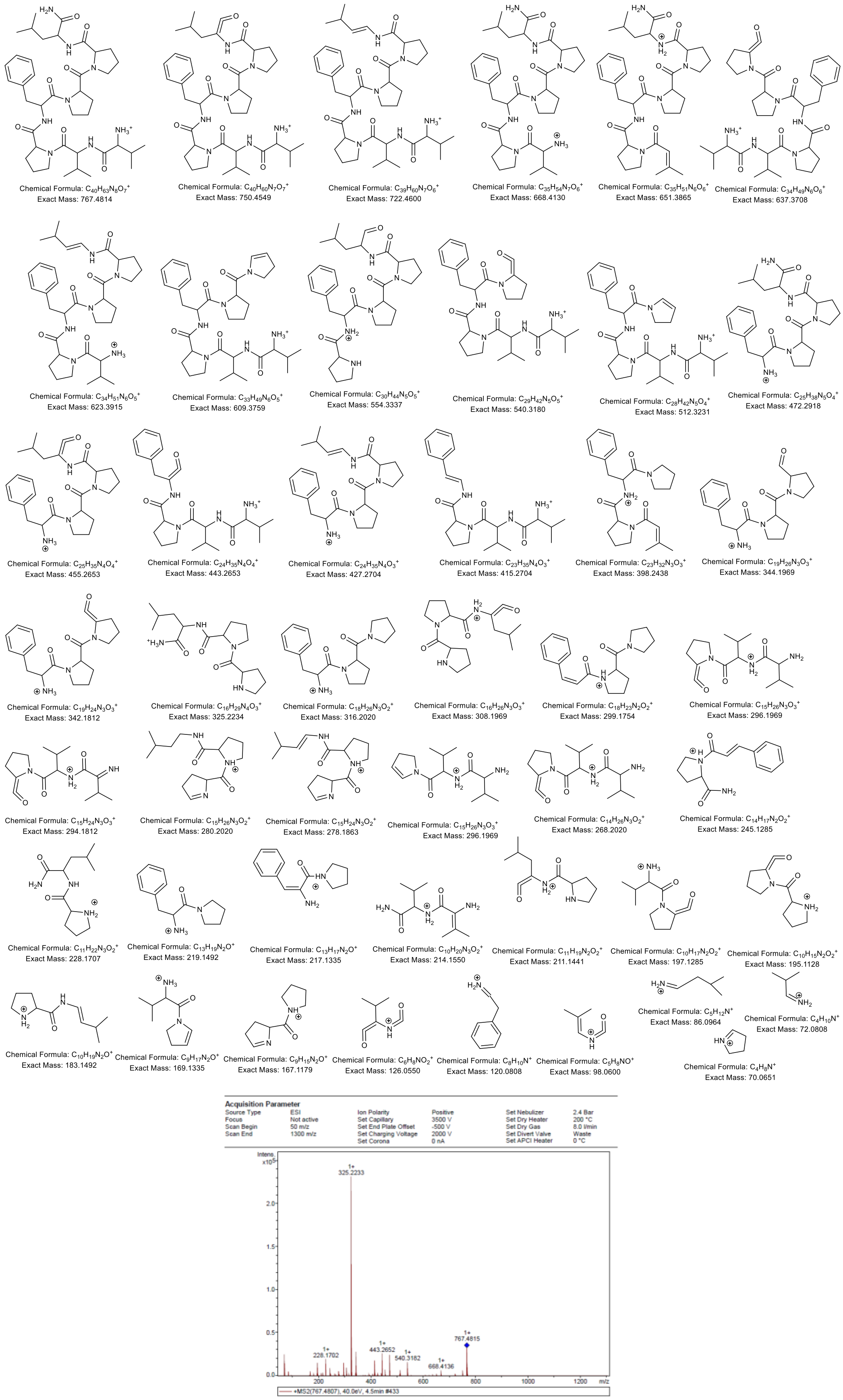

Table S5: Product ion spectra data for subarmigeride F (**6**) (*m/z* 768.4650 [M + H]+)

Product ion Assignment	(*m/z*)	Error, pm	Molecular Formula
Val-Val-Pro-Phe-Pro-Pro-Ile/Leu H+	768.4649	0.7	$C_{40}H_{62}N_7O_8$
Val-Val-Pro-Phe-Pro-Pro-Ile/Leu + H+	750.4536	1.7	$C_{40}H_{60}N_7O_7$
Val-Pro-Phe-Pro-Pro-Ile/Leu + H+	669.3976	-0.9	$C_{35}H_{53}N_6O_7$
Val-Val-Pro-Phe-Pro-Pro + H+	637.3698	1.6	$C_{34}H_{49}N_6O_6$
Val-Val-Pro-Phe-Pro-Pro + H+	609.3779	-3.2	$C_{33}H_{49}N_6O_5$
Val-Val-Pro-Phe-Pro + H+	540.3176	0.7	$C_{29}H_{42}N_5O_5$
Val-Val-Pro-Phe-Pro + H+	512.3231	0.1	$C_{28}H_{42}N_5O_4$
Phe-Pro-Pro-Ile/Leu + H+	473.2756	0.4	$C_{25}H_{37}N_4O_5$
Val-Val-Pro-Phe + H+	443.2655	-0.5	$C_{24}H_{35}N_4O_4$
Val-Val-Pro-Phe + H+	415.2704	0	$C_{23}H_{35}N_4O_3$
Val-Val-Pro-Phe + H+	413.2543	0.9	$C_{23}H_{33}N_4O_3$
Phe-Pro-Pro + H+	372.1927	-2.6	$C_{20}H26N_3O4$
Phe-Pro-Pro + H+	344.1965	-2.7	$C_{17}H_{24}N_6O_2$
Phe-Pro-Pro + H+	342.1808	-2.6	$C_{17}H_{22}N_6O_2$
Phe-Pro-Pro + H+	327.1707	-1.3	$C_{19}H_{23}N_2O_3$
Pro-Pro-Ile/Leu + H+	326.2072	0.8	$C_{16}H_{28}N_3O_4$
Phe-Pro-Pro + H+	316.2017	-3.5	$C_{16}H_{24}N_6O$
Pro-Pro-Ile/Leu + H+	308.1957	3.9	$C_{16}H_{26}N_3O_3$
Phe-Pro-Pro + H+	299.1739	4.9	$C_{18}H_{23}N_2O_2$
Val-Val-Pro + H+	296.1965	1.2	$C_{15}H_{26}N_3O_3$
Pro-Pro-Ile/Leu + H+	278.1857	2	$C_{15}H_{24}N_3O_2$
Val-Val-Pro + H+	268.2021	-0.5	$C_{14}H_{26}N_3O_2$
Phe-Pro + H+	245.1282	0.8	$C_{14}H_{17}N_2O_2$
Pro-Ile/Leu + H+	229.1542	2	$C_{11}H_{21}N_2O_3$
Phe-Pro + H+	219.1488	1.6	$C_{13}H_{19}N_2O$
Phe-Pro + H+	217.1328	3.5	$C_{13}H_{17}N_2O$
Val-Pro + H+	197.1277	3.6	$C_{10}H_{17}N_2O_2$
Pro-Pro + H+	195.1120	-3	$C_8H_{13}N_5O$
Val-Pro + H+	169.1329	3.9	$C_9H_{17}N_2O$
Ile/Leu immonium fragment + H+	86.0956	10	$C_5H_{12}N$
Val immonium fragment + H+	72.0803	6.2	$C_4H_{10}N$
Pro immonium fragment + H+	70.0649	3.9	C_4H_8N

Figure S16: Fragmentations pattern and positive ion mode high-resolution ESI MS/MS spectrum for subarmigeride F (**6**) (*m*/*z* 768.4650 [M + H]⁺)

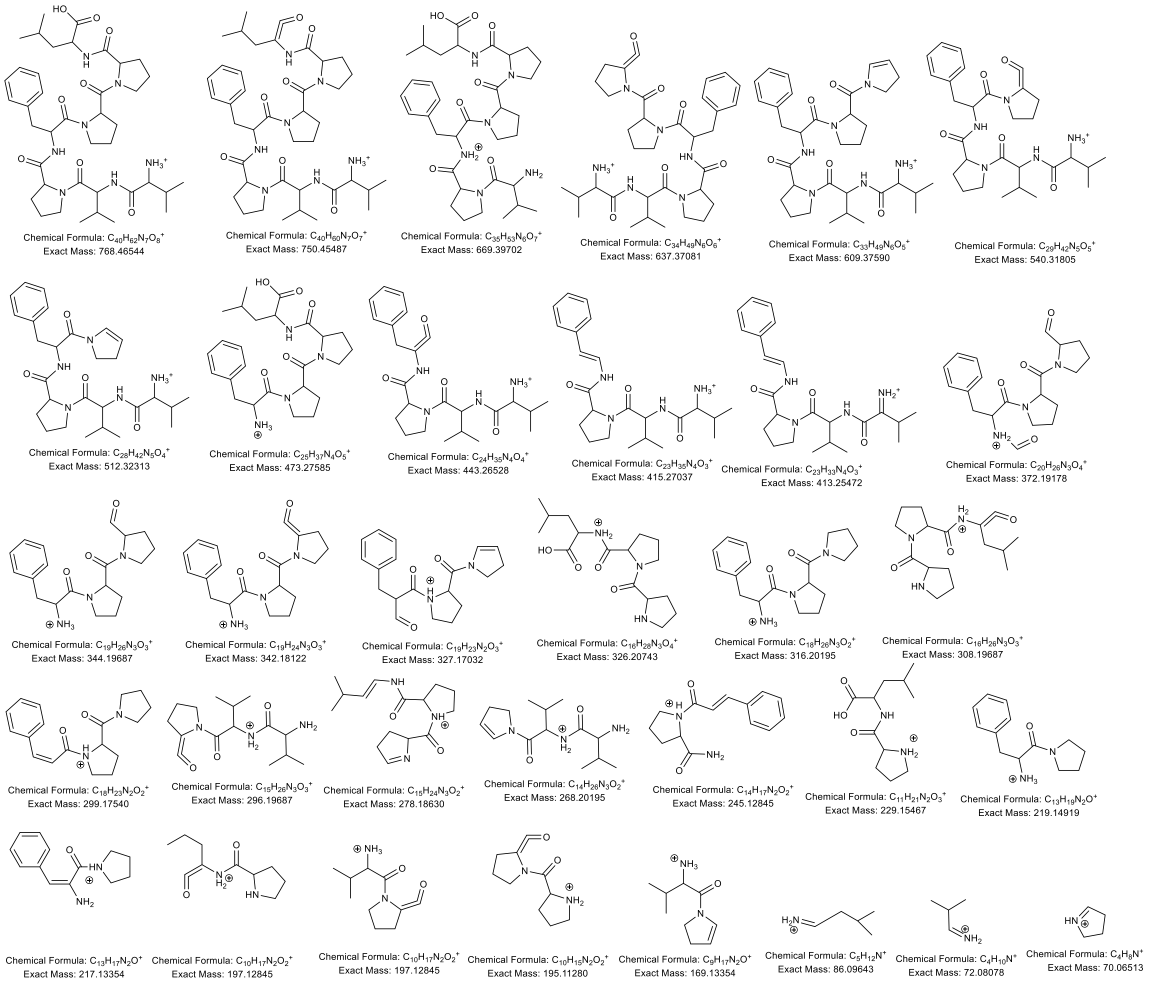

Acquisition Parameter

Source Type	ESI	Ion Polarity	Positive	Set Nebulizer	2.4 Bar
Focus	Not active	Set Capillary	3500 V	Set Dry Heater	200 °C
Scan Begin	50 m/z	Set End Plate Offset	-500 V	Set Dry Gas	8.0 l/min
Scan End	1300 m/z	Set Charging Voltage	2000 V	Set Divert Valve	Waste
		Set Corona	0 nA	Set APCI Heater	0 °C

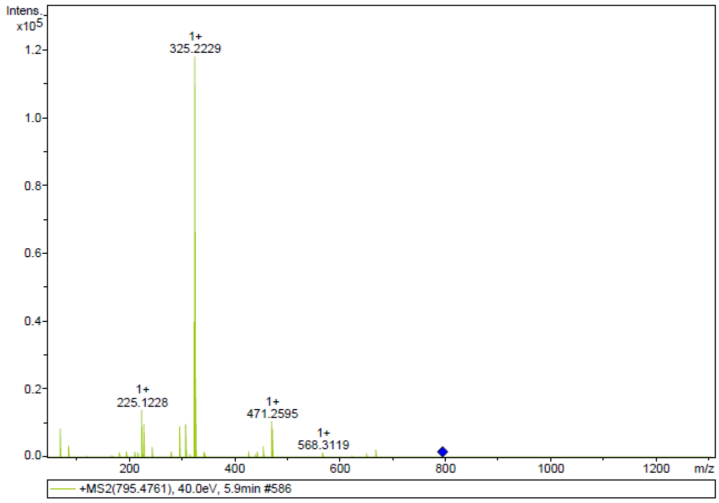

Table S6: Product ion spectra data for subarmigeride G (**7**) (*m*/*z* 938.5698 [M + H]$^+$)

Product ion Assignment	(*m*/*z*)	Error, pm	Molecular Formula
COOH-Pro-Val-Ile/Leu-Ile/Leu-Phe-Pro-Pro-Ile/Leu-NH_2 + H^+	938.5698	1.2	$C_{48}H_{76}N_9O_{10}$
Pro-Val-Ile/Leu-Ile/Leu-Phe-Pro-Pro-Ile/Leu-NH_2 + H^+	797.4908	1.5	$C_{41}H_{65}N_8O_8$
COOH-Pro-Val-Ile/Leu-Ile/Leu-Phe-Pro + H^+	711.4090	-1.9	$C_{37}H_{55}N_6O_8$
Pro-Val-Ile/Leu-Ile/Leu-Phe-NH_2 + H^+	585.3761	-0.3	$C_{31}H_{49}N_6O_5$
Phe-Pro-Pro-Ile/Leu-NH_2 + H^+	472.2919	0	$C_{25}H_{38}N_5O_4$
COOH-Pro-Val-Ile/Leu-Ile/Leu + H^+	467.2863	0.2	$C_{23}H_{39}N_4O_6$
Phe-Pro-Pro-Ile/Leu-NH_2 + H^+	455.2647	1.2	$C_{25}H_{35}N_4O_4$
COOH-Pro-Val-Ile/Leu-Ile/Leu + H^+	439.2915	-0.1	$C_{22}H_{39}N_4O_5$
Phe-Pro-Pro-Ile/Leu + H^+	427.2698	1.4	$C_{24}H_{35}N_4O_3$
COOH-Pro-Val-Ile/Leu + H^+	354.2021	0.6	$C_{17}H_{28}N_3O_5$
Phe-Pro-Pro + H^+	342.1804	2.5	$C_{19}H_{24}N_3O_3$
Pro-Pro-Ile/Leu + H^+	325.2231	0.9	$C_{16}H_{29}N_4O_3$
Pro-Pro-Ile/Leu + H^+	308.1966	0.9	$C_{16}H_{26}N_3O_3$
Pro-Pro-Ile/Leu + H^+	280.2016	1.3	$C_{15}H_{26}N_3O_2$
Pro-Ile/Leu + H^+	255.1335	1.8	$C_{12}H_{19}N_2O_4$
Phe-Pro + H^+	245.1281	1.6	$C_{14}H_{17}N_2O_2$
Pro-Ile/Leu-NH_2 + H^+	228.1706	0.4	$C_{11}H_{22}N_3O_2$
Pro-Ile/Leu + H^+	217.1336	-0.3	$C_{13}H_{17}N_2O$
Pro-Ile/Leu + H^+	211.1434	3.3	$C_{11}H_{19}N_2O_2$
Pro-Pro + H^+	195.1111	8.6	$C_{10}H_{15}N2O_2$
Pro-Ile/Leu + H^+	183.1485	3.9	$C_{10}H_{19}N_2O$
Ile/Leu immonium fragment + H^+	86.0958	7.3	$C_5H_{12}N$
Pro immonium fragment + H^+	70.0645	9.5	C_4H_8N

Figure S17: Fragmentations pattern and positive ion mode high-resolution ESI MS/MS spectrum for subarmigeride G (**7**) (*m/z* 938.5698 [M + H]⁺)

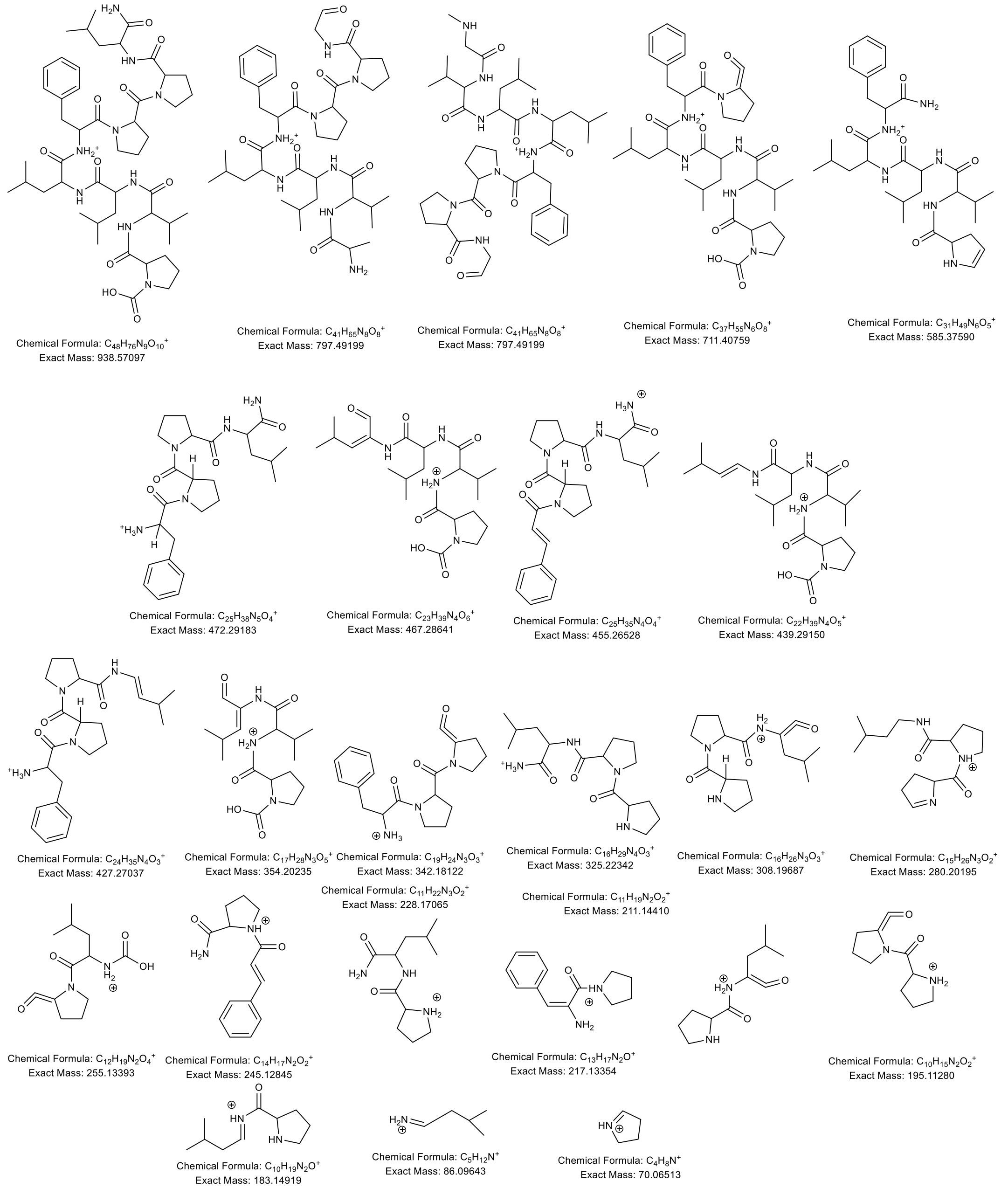

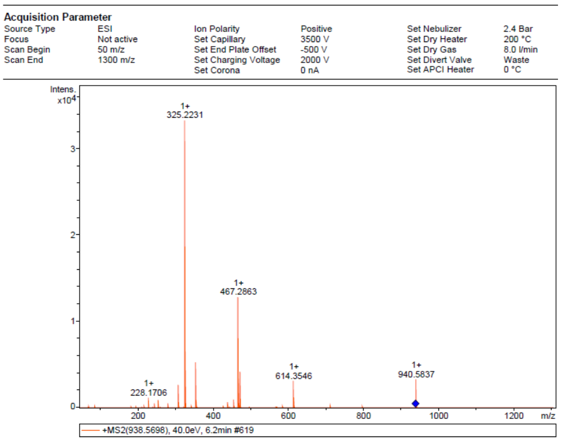

Table S7: Product ion spectra data for subarmigeride H (**8**) (*m/z* 795.4763 [M + H]$^+$)

Product ion Assignment	(*m/z*)	Error, pm	Molecular Formula
CHO-Val-Val-Pro-Phe-Pro-Pro-Ile/Leu-NH$_2$ + H$^+$	795.4761	0.3	$C_{41}H_{63}N_8O_8$
Val-Val-Pro-Phe-Pro-Pro-Ile/Leu-NH$_2$ + H$^+$	668.4117	1.9	$C_{35}H_{54}N_7O_6$
Val-Pro-Phe-Pro-Pro-Ile/Leu + H$^+$	623.3907	1.3	$C_{34}H_{51}N_6O_5$
Phe-Pro-Pro-Ile/Leu-NH$_2$ + H$^+$	472.2911	1.5	$C_{25}H_{38}N_5O_4$
Val-Val-Pro-Phe + H$^+$	471.2595	1.4	$C_{25}H_{35}N_4O_5$
Phe-Pro-Pro-Ile/Leu + H$^+$	455.2648	1.1	$C_{25}H_{35}N_4O_4$
CHO- Val-Val-Pro-Phe + H$^+$	443.2644	1.9	$C_{24}H_{35}N_4O_4$
Phe-Pro-Pro + H$^+$	344.1961	2.1	$C_{19}H_{26}N_3O_3$
Phe-Pro-Pro + H$^+$	327.1704	-0.3	$C_{19}H_{23}N_2O_3$
Pro-Pro-Ile/Leu-NH$_2$ + H$^+$	325.2229	1.6	$C_{16}H_{29}N_4O_3$
CHO-Val-Val-Pro + H$^+$	324.1913	1.5	$C_{16}H_{26}N_3O_4$
Phe-Pro-Pro + H$^+$	316.2017	0.8	$C_{18}H_{26}N_3O_2$
Pro-Pro-Ile/Leu + H$^+$	308.1964	1.5	$C_{16}H_{26}N_3O_3$
Val-Val-Pro + H$^+$	296.1964	1.5	$C_{15}H_{26}N_3O_3$
Phe-Pro + H$^+$	245.1278	2.5	$C_{14}H_{17}N_2O_2$
Pro-Ile/Leu-NH$_2$ + H$^+$	228.1701	2.3	$C_{11}H_{22}N_3O_2$
Val-Val + H$^+$	225.1228	2.3	$C_{11}H_{17}N_2O_3$
Pro-Phe + H$^+$	217.1329	2.8	$C_{13}H_{17}N_2O$
Pro-Ile/Leu + H$^+$	211.1433	4	$C_{11}H_{19}N_2O_2$
Val-Pro + H$^+$	197.1274	5.6	$C_{10}H_{17}N_2O_2$
Pro-Ile/Leu + H$^+$	183.1479	6.9	$C_{10}H_{19}N_2O$
Val-Pro + H$^+$	169.1330	3.5	$C_9H_{17}N_2O$
Phe immonium fragment + H$^+$	120.0814	-5	$C_8H_{10}N$
Ile/Leu immonium fragment + H$^+$	86.0962	3.1	$C_5H_{12}N$
Pro immonium fragment + H$^+$	70.0647	5.9	C_4H_8N

Figure S18: Fragmentations pattern and positive ion mode high-resolution ESI MS/MS spectrum for subarmigeride H (**8**) (*m/z* 795.4763 [M + H]+)

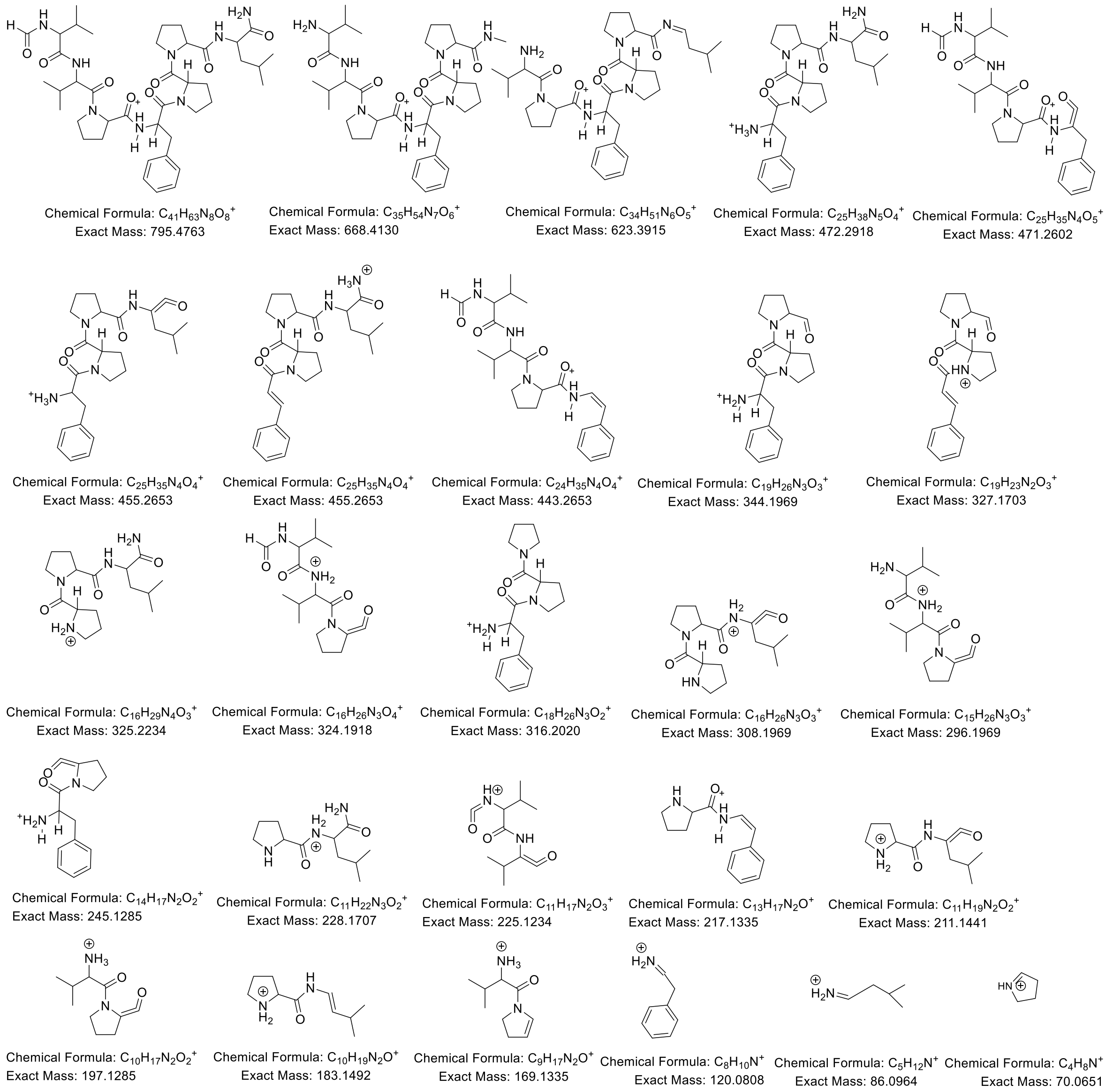

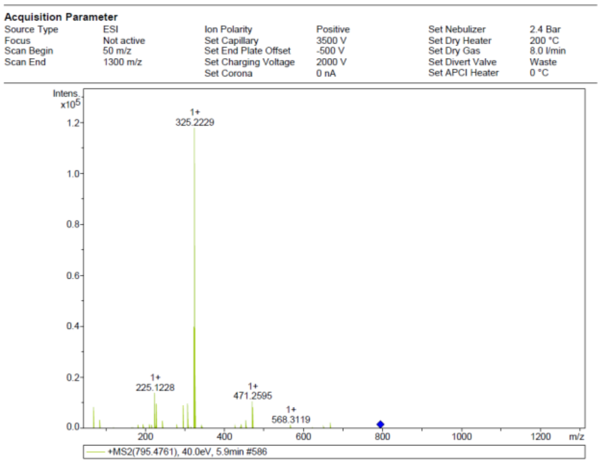

Figure S19: Comparison of MS/MS spectra of the feature *m/z* 857.4920 at 6.036 min in the cyanobacterial strain PMC 1052.18 (*Spirulina* sp.) from a mangrove in Guadeloupe (A) and the feature *m/z* 857.4909 at 5.934 min in the marine sponge *C. subarmigera* (B). Comparison of extracted ion chromatograms for *m/z* 857.4912 (tolerance 10 ppm) from the crude extracts of the cyanobacterial strain PMC 1052.18 (C) and the marine sponge *C. subarmigera* (D).

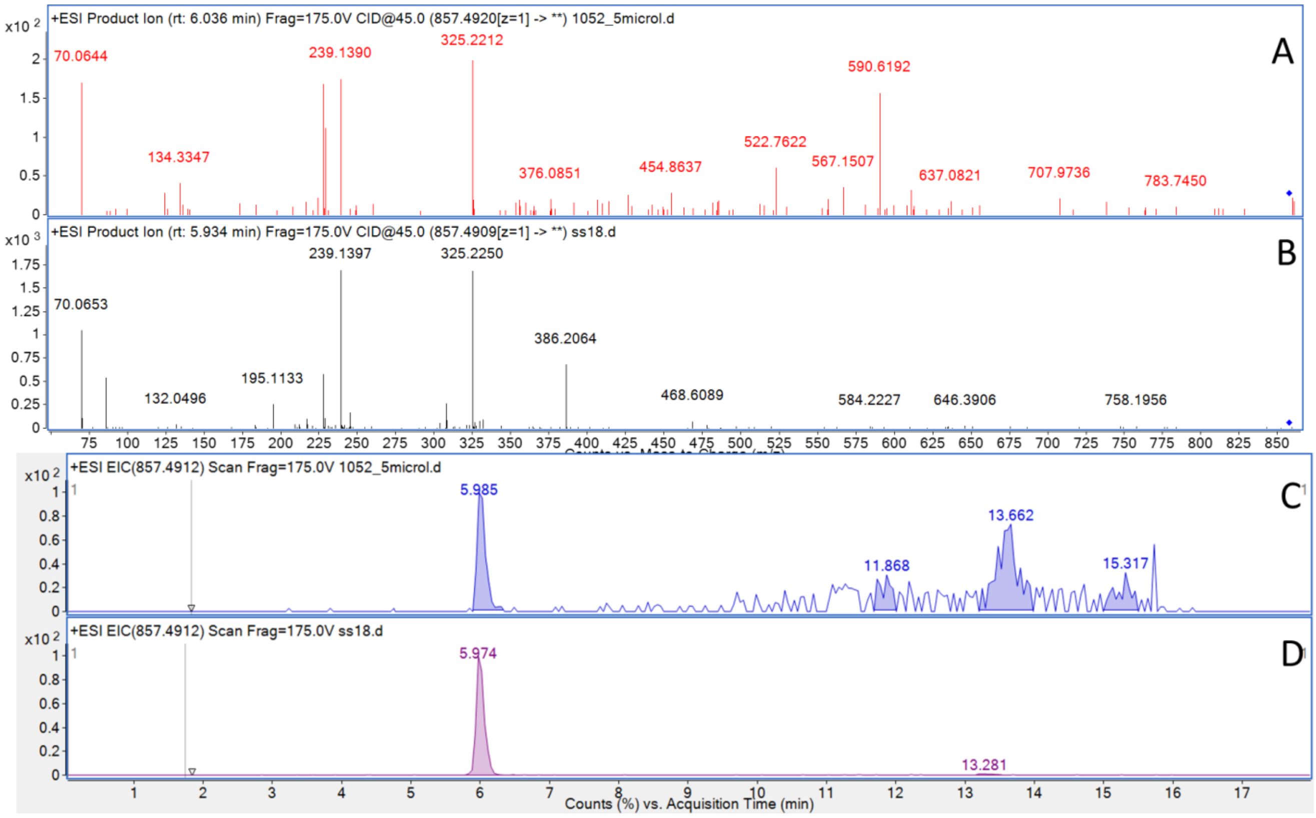

Figure S20: Helically coiled morphology of *Spirulina* sp. PMC 1052.18.

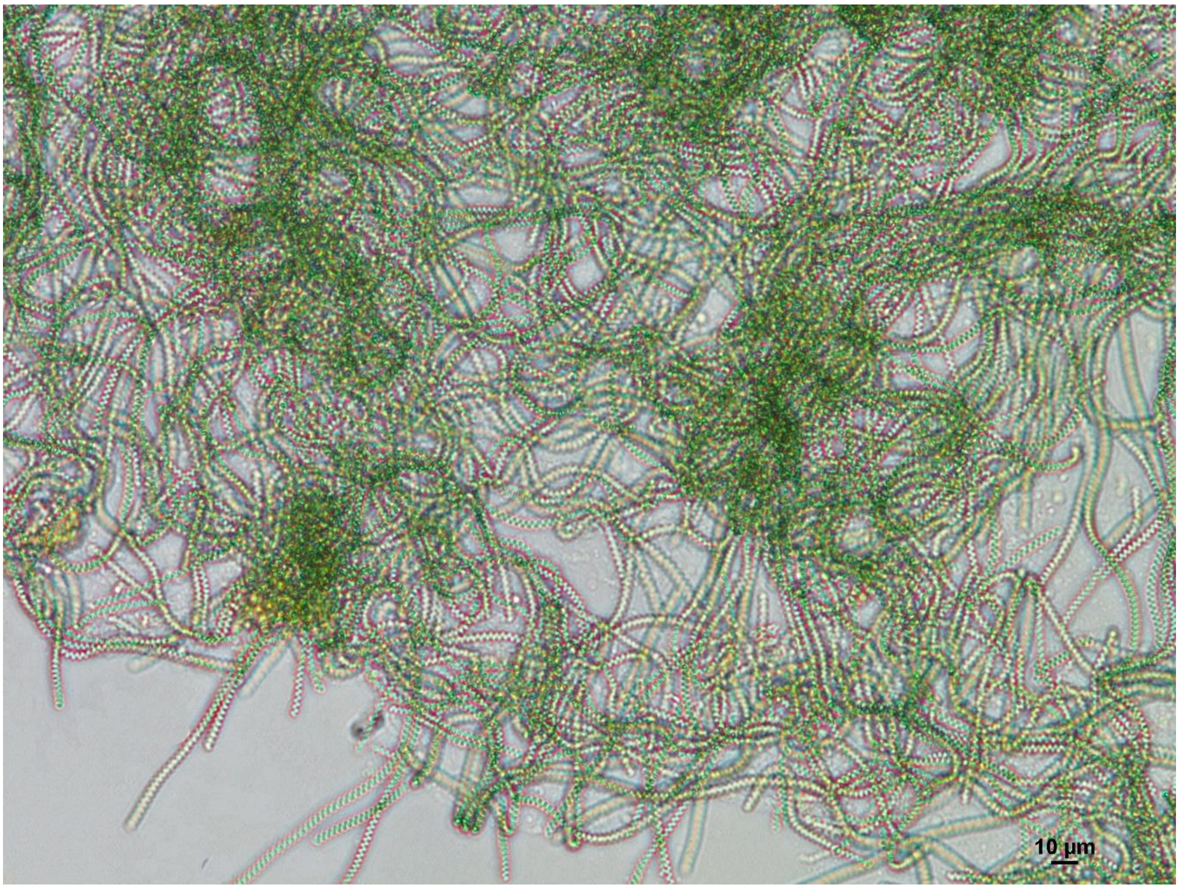

Figure S21: Molecular networks obtained using the Feature-Based Molecular Network workflow on GNPS (https://gnps.ucsd.edu/ProteoSAFe/status.jsp?task=8a40068370b44e21855c1e14647ff23a)

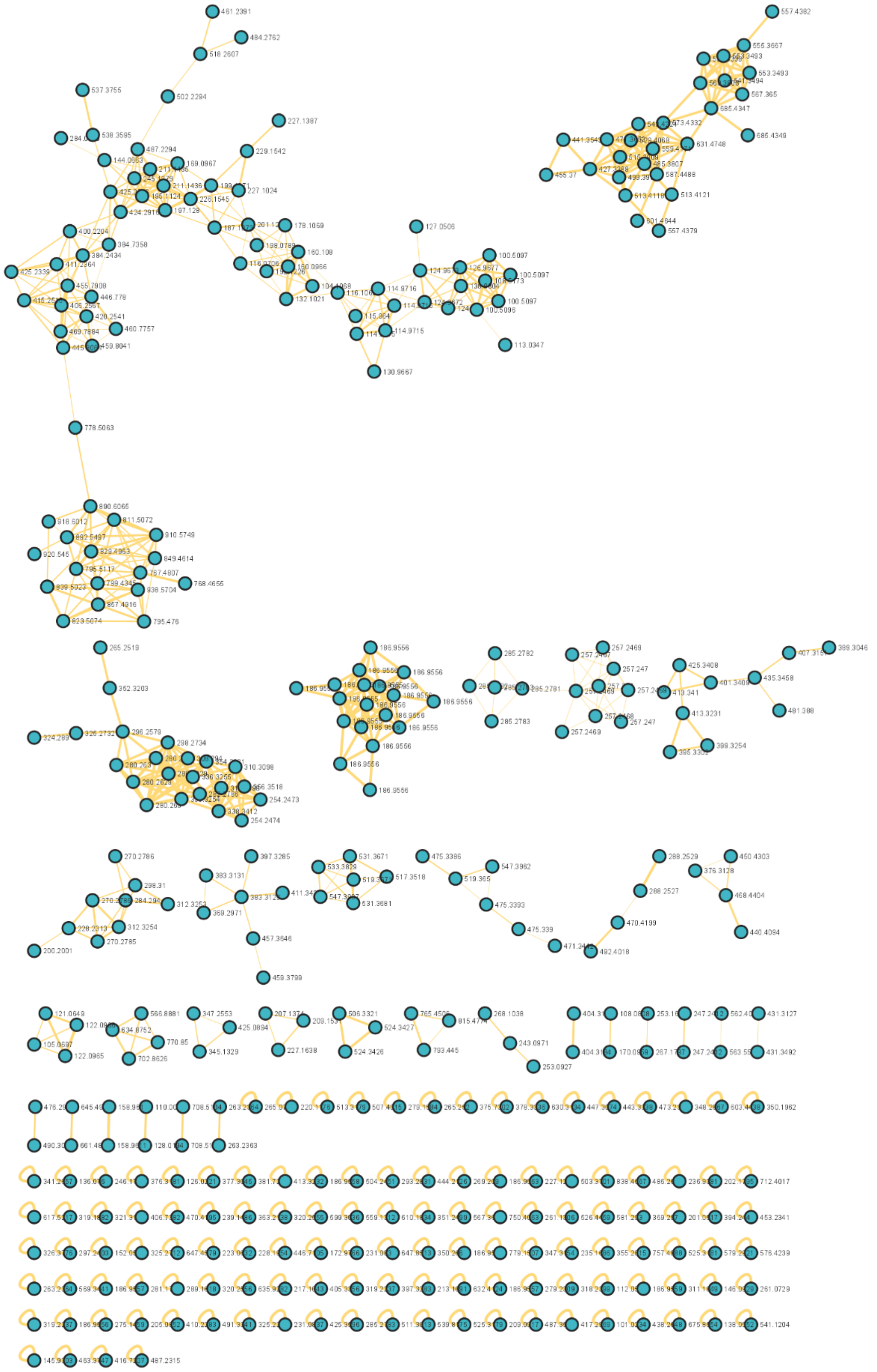

Supplement: Supplementary file 1 [file marinedrugs-20-00673-s001.zip › marinedrugs-1960037-supplementary.pdf]
